# *Chloride intracellular channel* gene knockdown induces insect cell lines death and level increases of intracellular calcium ions

**DOI:** 10.3389/fphys.2023.1217954

**Published:** 2023-07-06

**Authors:** Jiqiang Song, Yanping Yu, Zhichao Yan, Shan Xiao, Xianxin Zhao, Fang Wang, Qi Fang, Gongyin Ye

**Affiliations:** ^1^ State Key Laboratory of Rice Biology and Breeding & Ministry of Agricultural and Rural Affairs Key Laboratory of Molecular Biology of Crop Pathogens and Insects, Institute of Insect Sciences, Zhejiang University, Hangzhou, China; ^2^ Department of Entomology, Nanjing Agricultural University, Nanjing, China

**Keywords:** insect cell lines, chloride intracellular channel, RNA interference, cell death, intracellular calcium ions

## Abstract

Chloride intracellular channel (CLIC) is a member of the chloride channel protein family for which growing evidence supports a pivotal role in fundamental cellular events. However, the physiological function of CLIC in insects is still rarely uncovered. The ovary-derived High Five (Hi-5) cell line isolated from the cabbage looper (*Trichoplusia ni*) is widely used in laboratories. Here, we studied both characteristics and functions of CLIC in Hi-5 cells (*Tn*CLIC). We identified the *TnCLIC* gene in Hi-5 cells and annotated highly conserved CLIC proteins in most insect species. After RNA interference of *TnCLIC*, the phenomenon of significantly increased cell death suggests that the *Tn*CLIC protein is essential for the survival of Hi-5 cells. The same lethal effect was also observed in *Spodoptera frugiperda* 9 and *Drosophila melanogaster* Schneider 2 cells after *CLIC* knockdown. Furthermore, we found that this kind of cell death was accompanied by increases in intracellular calcium ions after *TnCLIC* knockdown with the transcriptomic analyses and the detection of calcium levels. Our results provide insights into insect CLIC as a key factor for cell survival and lay the foundation for the cell death mechanism.

## 1 Introduction

The cabbage looper moth *Trichoplusia ni* (Lepidoptera: Noctuidae) is a destructive agricultural pest (Chen et al., 2019). Its caterpillars defoliate crucifer plants, leading to serious production losses (Shorey et al., 1962). The High Five (Hi-5) cell line is isolated from the ovarian germ cells of *T. ni* (Hink, 1970). It is an insect cell line with widespread application in industry and academic laboratories, such as baculovirus-mediated recombinant protein expression (Wickham and Nemerow, 1993). Put simply, codon usage analysis proved that Hi-5 cells were robustly capable of expressing proteins, and how to optimize the expression level of some glycosylated, secretory and membrane proteins was provided (Yu et al., 2016). With the development of sequencing technology, the genome of the Hi-5 cell line has been sequenced and assembled (Fu et al., 2018). Nonetheless, fundamental biological research based on the Hi-5 cell line is still poorly understood.

The model of subtle ion permeation lays the groundwork for many physiological processes (Gouaux and Mackinnon, 2005). Therefore, the potential role of ion channels is involved in the maintenance of cellular homeostasis (Gadsby, 2009). Given the recent advances in cryo-EM technology, the structure of chloride channels (CLCs) in an increasing number of species is being solved and provides a foundation to further dissect the regulatory mechanism (Park et al., 2017). For instance, a proton-activated chloride channel (PAC) was reported to be active in acid-induced cell death (Yang et al., 2019; Ruan et al., 2020). Additionally, *CLCN2*, encoding a voltage-gated chloride channel with mutations, was found to establish the cause of a substantial fraction of aldosterone production and hypertension (Scholl et al., 2018). These findings support that CLCs have diverse biological functions under both physiological and pathological conditions. However, it is noteworthy that the structure of insect CLCs has rarely been reported by comparison with other ion channels.

More recently, chloride intracellular channel (CLIC), a class of intracellular organelle ion channels, has attracted great attention in many species (Ashley, 2003). For mammals, the CLIC superfamily (CLIC1-6) mainly regulates a variety of cellular events, including cell differentiation, apoptosis and metastatic competence (Argenzio and Moolenaar, 2016; Wang et al., 2021). Growing evidence also supports the role of mammalian CLIC1-6 in different diseases (Leanza et al., 2013), especially CLIC1 (Francisco et al., 2020) and CLIC4 (Chuang et al., 2022). In addition, dehydroascorbate reductase (DHAR) in plants is appreciably homologous to animal CLIC (Elter et al., 2007) and has been reported an association with glutathione homeostasis for plant defense, growth and development (Ding et al., 2020). As to *Caenorhabditis elegans*, EXC-4 provides a vital insight into CLIC function because it is a primordial player for tubulogenesis in vascular development (Arena et al., 2022). These findings correspond to a controversial issue with CLIC proteins that may possess other cellular functions in addition to their proposed roles as chloride channels (Littler et al., 2010). In contrast to chloride channels on the plasma membrane, CLIC proteins are more difficult to study and have been overlooked thus far due to technical limitations (Chen et al., 2017).

RNA interference (RNAi) is applicated to the study of ion channel physiology in animals (Palmer et al., 2006). For example, small interfering RNA (siRNA) was used to knock down the *TASK-1* potassium channel gene (Gurney and Hunter, 2005). A dose-dependent effect was observed for the mortality of *Tribolium castaneum* by injection and oral delivery of dsRNA, targeting potassium ion channels (Alshukri et al., 2019) or the voltage-gated sodium ion channel (VGSC) (Abd El Halim et al., 2016). In addition, knocking down *CLIC* expression in *Drosophila* modulated ethanol sedation sensitivity by the oxidation-reduction mechanism (Weston et al., 2021). However, to date, few functional studies of the CLIC protein in insect cells have been reported. Here, we identified conserved CLIC proteins in numerous insects and verified the cell death phenotype in Hi-5, *Spodoptera frugiperda* 9 (Sf9) and *Drosophila melanogaster* Schneider 2 (S2) cells after RNAi experiments. Transcriptomic analyses and the measurement of calcium levels showed that this kind of cell death involved increases in intracellular calcium ions after *TnCLIC* knockdown. Our results suggest that *Tn*CLIC is an essential protein for the survival of insect cells and the cell death is accompanied by level increases of intracellular calcium ions.

## 2 Materials and methods

### 2.1 Cell culturing

Insect *Trichoplusia ni* High Five (Hi-5) cells were cultured in an incubator at 27°C using Grace’s Insect Medium with 10% fetal bovine serum (WISENT, Nanjing, China) following the manufacturer’s instructions. Analogously, *S. frugiperda* 9 (Sf9) cells were cultured using Sf-900™ II SFM and *D. melanogaster* Schneider 2 (S2) cells were cultured using Schneider’s *Drosophila* Medium at 27°C. Furthermore, human embryonic kidney 293T (HEK 293T) cells were grown in Dulbecco’s modified Eagle’s medium at 37°C and 5% CO_2_ containing 10% FBS and penicillin‒streptomycin. These cultured media were purchased from Life Technologies Corporation.

### 2.2 Phylogenetic analysis and multiple sequence alignment

Raw data on the genome, transcript, and protein sequences were retrieved from the NCBI and InsectBase 2.0 (Mei et al., 2022). To identify homologous genes in different species, tBLASTn analysis was performed using the *Trichoplusia ni* chloride intracellular channel (*Tn*CLIC) protein sequence as a query (e-value < 1e-5). Extracted contigs were predicted using the SoftBerry FGENESH program (Solovyev et al., 2006) and manually checked by comparing them in the GenBank database. The SignalP 5.0 server was used for signal peptide prediction (Almagro Armenteros et al., 2019). Collected homologous protein sequences from 110 insect species were aligned using MAFFT (Katoh and Standley, 2013) and trimmed using TrimAL (Capella-Gutierrez et al., 2009). Then, maximum-likelihood phylogeny was calculated using IQ-TREE (version 2.0.3) (Nguyen et al., 2015) under the LG + G4 substitution model with 1, 000 ultrafast bootstraps. The phylogenetic tree was then optimized and visualized via iTOL (Letunic and Bork, 2021). For multiple sequence alignment, protein sequences of *Tn*CLIC, *Sf*CLIC, *Dm*CLIC, *Nl*CLIC, *Pp*CLIC, *Tc*CLIC, Exc-4, and human CLIC4 were aligned using ggmsa R package (Zhou et al., 2022) and the consensus sequence logo was generated to show the frequency of amino acids (https://github.com/YuLab-SMU/supplemental-ggmsa).

### 2.3 Gene cloning and construction of recombinant plasmids

Total RNA was extracted from cells using TRIzol reagent (Invitrogen, CA, United States) according to the manufacturer’s protocol. Then, RNAs were reverse-transcribed into single-stranded complementary DNAs (cDNAs) using the *TransScript*® One-Step gDNA Removal and cDNA Synthesis SuperMix (TransGen Biotech, Beijing, China). Cloned PCR amplifications with restriction site sequences (KpnI and XbaI) of *TnCLIC* were carried out using KOD One™ PCR Master Mix (TOYOBO, Shanghai, China). Primers were designed by CE Design (Vazyme, Nanjing, China) and are listed in Supplementary Table S1. Afterward, pCold-I plasmids digested by FastDigest KpnI and XbaI (Thermo Fisher, CA, United States) were recombined with PCR products using the ClonExpress Ultra One Step Cloning Kit (Vazyme, Nanjing, China). After sequencing, the recombinant plasmid was transformed into BL21 Chemically Competent Cell (TransGen, Beijing, China).

### 2.4 Prokaryotic expression and antibody preparation

To induce protein expression, 1 mM isopropyl-β-d-thiogalactoside (IPTG) was added when the OD value of the bacterial culture (grown at 37°C) reached 0.5–0.6. After 24 h of incubation with 120 rpm shaking at 15°C, the recombinant *E. coli* cells were harvested by centrifugation and lysed with BugBuster® Master Mix (Novagen, MA, United States) at 4°C. Since the pCold-I vector is equipped with a 6 × His tag, recombinant proteins were purified by His-tag affinity chromatography using cOmplete His-Tag Purification Resin and Ni-NTA BUFFER KIT (Merck, United States) according to the manufacturer’s instructions. The purified proteins were then desalted using Zeba™ Spin Desalting Columns, 7K MWCO, 2 mL (Thermo Scientific, Waltham, United States). SDS‒PAGE and western blot analyses were performed after the protein concentration was determined using Quick Start Bradford Dye Reagent (Bio-Rad, CA, United States) (Bradford, 1976). The mouse monoclonal antibody (a label-free primary antibody) against 6 × His tag was purchased from GenScript (Nanjing, China). Finally, the *Tn*CLIC antibody was a rabbit polyclonal antibody made by HuaAn Biotechnology Co., Ltd. (Hangzhou, China) using the purified protein.

### 2.5 siRNA transfection

Before siRNA knockdown experiments, cells were plated and grown until 60%–80% confluence. The next day, cells were transfected with siRNA (100 nM final concentration) using Lipofectamine™ RNAiMAX Reagent (Invitrogen, CA, United States) in OptiMEM medium (Life Technologies Corporation, NY, United States) following the manufacturer’s instructions. Two pairs of siRNA per gene were designed and synthesized by Sangon Biotech (Shanghai, China). The siRNA sequences from 5′ to 3′ were as follows: siRNA-*Tn*CLIC-1 and siRNA-*Sf*CLIC-1: GCA​AGU​ACU​UCG​UCG​ACU​UTT; siRNA-*Tn*CLIC-2: GCC​UCG​CAA​UAC​UGG​AGA​ATT; siRNA-*Sf*CLIC-2: GCA​GAG​GGA​CUA​GAU​UCU​UTT; siRNA-*Dm*CLIC-1: GCA​AGU​ACU​UUG​UCG​ACU​UTT; siRNA-*Dm*CLIC-2: CCA​CGU​UUA​CCA​CAU​ACA​UTT; siRNA-NC: UUC​UCC​GAA​CGU​GUC​ACG​UTT. Detailed information is listed in Supplementary Table S1.

### 2.6 Real-time quantitative PCR

For real-time quantitative PCR (RT‒qPCR) analysis, samples were collected at different time post-transfection. The related reaction was conducted with the CFX 96 Real-Time Detection System (Bio-Rad, CA, United States) using ChamQ SYBR qPCR Master Mix (without ROX) (Vazyme, Nanjing, China). Program conditions for thermal cycling were 95°C for 30 s, 40 cycles of 95°C for 5 s, and 60°C for 30 s. Three biological replicates were used for each treatment. Ct values were normalized to *T. ni* ribosomal protein S5, *S. frugiperda* actin, *D. melanogaster* ribosomal protein L32 and *Homo sapiens* GAPDH genes, respectively. The relative expression was calculated using the 2^-△△Ct^ method (Livak and Schmittgen, 2001). Primers were designed on Primer3web, and their sequences are listed in Supplementary Table S1.

### 2.7 Western blotting

Knockdown samples were collected and lysed with I-PER Insect Cell Protein Extraction Reagent (Thermo Fisher Scientific, MA, United States) at 48 h. The total protein concentration was quantified to the same amount before loading onto an 8%–16% SDS‒PAGE gel. For each sample, 4 × protein loading buffer was added, boiled (100°C, 10 min), and cooled in an ice bath for 10 min. Then, the separated proteins were transferred to a polyvinylidene difluoride (PVDF) membrane (Sigma, United States). The primary antibodies used were a rabbit anti-*Tn*CLIC antibody and a mouse anti-β-actin antibody (diluted 1:2, 000). For the corresponding secondary antibodies, a goat anti-rabbit or goat anti-mouse IgG-horseradish peroxidase (HRP) conjugate was subsequently incubated (diluted 1:2, 000). The *Tn*CLIC polyclonal antibody was prepared (HuaAn, Hangzhou, China) as above, and other antibodies were purchased commercially (GenScript, Nanjing, China). The chemiluminescence signal was developed using the Super Signal West Dura Extended Duration Substrate (Thermo Scientific, United States) and detected with a UVP Chemi Doc-It Imaging System (Upland, United States). Each experiment involved three biological replicates and the integrated density for each band was measured with Image J software (http://imagej.nih.gov/ij/, version 1.53).

### 2.8 Cell viability assay

Cell viability was measured using the RealTime-Glo™ MT Cell Viability Assay (Promega; Madison, WI, United States) according to the manufacturer’s instructions (Krall et al., 2021). The kit determines the number of viable cells in culture by measuring the reducing potential of cells and metabolism (MT); thus, the chemiluminescent signal correlates with the number of viable cells. In summary, the MT Cell Viability Substrate and NanoLuc® Enzyme were allowed to equilibrate to 37°C in a water bath before use. Then, both reagents were added to the cell suspension to a final concentration of 1X. After evenly mixing, cells were seeded on a 384-well assay plate at an equal density in a total volume of 70 μL per well and incubated at 27°C overnight. On the following day, 60%–80% confluent cells were transfected with siRNA at 100 nM final concentration as above. A Thermo Scientific Varioskan Flash (Thermo Scientific, Vantaa, Finland) was used to monitor luminescence at 48 h. Three replicates were performed for each treatment.

### 2.9 Cell death assay

Cell death was evaluated by measuring the fluorescence intensity of the CellTox Green Cytotoxicity Assay (Promega; Madison, WI, United States) following the manufacturer’s protocol (Krzyzosiak et al., 2018). Fluorescence intensity correlates with the loss of cell membrane integrity caused by cell death. The final dilution of CellTox™ Green Dye (a proprietary asymmetric cyanine dye) to 1:1, 000 was provided, and for more detailed experimental procedures see the description above for the measurement of cell viability. Hi-5 cell morphology was observed using a Leica DMi8 (Leica, Wetzlar, Germany) after transfection at 96 h. To avoid the influence of the cell medium on the fluorescent value, the mix was removed before measuring fluorescence with a microplate reader at 485 nm excitation and 525 nm emission. Three independent replicates for each treatment condition were performed.

### 2.10 Transcriptome sequencing and analysis

RNA extraction was conducted as described above. A total amount of 1 μg RNA per sample was used as input material for the RNA sample preparations. Sequencing libraries were generated using the TruSeq RNA Library Preparation Kit (Illumina, United States), and index codes were added to attribute sequences to each sample. The clustering of the index-coded samples was performed on a cBot Cluster Generation System using TruSeq PE Cluster Kit v3-cBot-HS (Illumina) according to the manufacturer’s instructions. After cluster generation, the library preparations were sequenced on an Illumina Novaseq platform, and 150 bp paired-end reads were generated. To ensure the quality of the information analysis, the raw reads were filtered by fastp software (https://github.com/OpenGene/fastp). The main steps of data filtering were as follows: 1) remove reads with adaptors; 2) remove reads with an N content of more than 10%; and 3) remove reads with low-quality bases (quality value <20) accounting for more than 50% of the total bases. FastQC (http://www.bioinformatics.babraham.ac.uk/projects/fastqc) was then used for quality control of clean data. Transcripts were aligned to the reference *Trichoplusia ni* genome using HISAT2 (Kim et al., 2015). Gene expression levels were normalized by calculating fragments per kilobase of transcript per million mapped reads (FPKM), and a value of 0.1 was used as a threshold for expressed genes (high, FPKM ≥15; moderate, 3.75 ≤ FPKM <15; low, 0.1 ≤ FPKM <3.75). StringTie was used to estimate the expression levels of the detected genes (Pertea et al., 2015). Gene differential expression analysis was performed using DESeq2 software (Love et al., 2014). Genes with a false discovery rate (FDR) below 0.05 and a fold change ≥2 were considered differentially expressed genes (DEGs). DEGs were then subjected to enrichment analysis for GO functions and KEGG pathways with *p*-adjust (FDR) < 0.05 as the significance threshold using clusterProfiler software (https://github.com/GuangchuangYu/clusterProfiler). This work was performed by Wuhan GrandOmics Biotechnology Co., Ltd.

### 2.11 Measurement of calcium concentration

The calcium concentration was detected using a Calcium Colorimetric Assay kit (Beyotime, Shanghai, China) according to the manufacturer’s operating manual (Ni et al., 2021). Briefly, Hi-5 cells were collected and washed with PBS after siRNA transfection at 0, 12, 24, 48, and 96 h. The precooled lysate was added to the sample and fully lysed on a rotating platform at 4°C for 15 min. Then, the supernatant was carefully aspirated after centrifuging at 12, 000 × g for 5 min. A ready-to-use working solution (75 μL chromogenic reagent and 75 μL calcium assay buffer) was added to the 50 μL cell lysates, followed by a 10 min incubation at 37°C in the dark. Finally, a 96-well microplate reader was used to measure the absorbance at 575 nm. The standard curve for calcium content was developed according to the different gradients of the standard solution to calculate the calcium concentration of each sample.

### 2.12 Detection of intracellular calcium ion levels

Intracellular calcium ion levels were determinated using a Fluo-4 Calcium Assay Kit (Beyotime, Shanghai, China) according to the manual instructions (Huang et al., 2021). The cell culture and siRNA transfection were performed as described above. After each specified treatment time of incubation, Hi-5 cells were loaded with Fluo-4 AM dye and solubility enhancer (1:500) in assay buffer at 27°C in the dark for 30 min. Then, Fluo-4 staining solution was removed gently and corresponding fluorescence values (490 nm excitation, 525 nm emission) were recorded immediately using a microplate reader. The experiment was performed independently three times.

### 2.13 Ca^2+^ chelators treatment

To chelate cytosolic Ca^2+^ signal transduction, Hi-5 cells were preloaded with BAPTA-AM (Aladdin, Shanghai, China) or EGTA-AM (AAT Bioquest, CA, United States) at 10 μM for 2 h in advance. After treatment, Hi-5 cells were transfected with siRNA as mentioned above. After 96 h, cell death was evaluated by CellTox™ Green Dye following the manufacturer’s protocol.

### 2.14 Mitochondrial membrane potential and reactive oxygen species assays

Intracellular mitochondrial membrane potential (MMP) signals were measured using a Mitochondrial Membrane Potential Assay Kit with Rhodamine 123 (Beyotime, Shanghai, China) (Qu et al., 2020), and reactive oxygen species (ROS) levels were assayed by a Reactive Oxygen Species Assay Kit (Beyotime, Shanghai, China) (Xin et al., 2022). In brief, Hi-5 cells were plated, maintained and transfected for 96 h. For the MMP assay, Hi-5 cells were washed with PBS after discarding the culture medium. Then, the cells were incubated with the working staining solution containing the cationic fluorescent probe 2-(6-amino-3-imino-3H-xanthen-9-yl) benzoic acid methyl ester (Rhodamine 123; 1:1, 000) at 37°C for 30 min. After discarding the supernatant, cell fluorescence was immediately detected at excitation (507 nm) and emission (529 nm) wavelengths. Hi-5 cells treated with 10 μM carbonyl cyanide 3-chlorophenylhydrazone (CCCP) for 20 min were used as a positive control. For intracellular ROS levels, Hi-5 cells were incubated with 2, 7-dichlorodihydrofluorescein diacetate (DCFH-DA; 1:1, 000) at 37°C for 20 min, which is easily oxidized to fluorescent dichlorofluorescein (DCF) by intracellular ROS. Hi-5 cells treated with a ROS-positive inducer (Rosup; 1:1, 000) for 30 min were used as a positive control. The fluorescence was detected at an excitation wavelength of 488 nm and at an emission wavelength of 525 nm after removing the supernatant.

### 2.15 Caspase 3/7 activity assay

Caspase 3/7 activation was measured using a Caspase-Glo® 3/7 Assay (Promega; Madison, WI, United States) according to the manufacturer’s protocol (Wang et al., 2017). Briefly, Hi-5 cells were grown in 96-well plates and treated with siRNA for 96 h. The ready-to-use reagent was prepared by swirling the buffer and substrate until the substrate was thoroughly dissolved. Afterward, an equal volume of reagent was added to each well and gently mixed. After incubation at room temperature for 30 min, the luminescence signal was immediately quantified using a microplate reader.

### 2.16 Cellular immunofluorescence

Hi-5 cells were seeded into a µ-Slide 4 Well (ibidi, Gräfelfing, Germany). The mitochondria and endoplasmic reticulums (ERs) in the cells were stained with 100 nM MitoTracker™ Red CMXRos (Thermo Fisher, CA, United States) and ER-Tracker Red (Beyotime, Shanghai, China) for 30 min, respectively. After washing with PBS quickly, the cells were fixed with 4% paraformaldehyde (PFA) at room temperature for 10 min. The samples were subsequently washed with PBS three times for 10 min each, permeabilized with 0.01% Triton X-100 for 15 min, blocked with 5% bovine serum albumin (BSA) for 1 h at room temperature, and incubated with primary antibodies overnight at 4°C. The next day, after three washes with PBST (0.1% Tween-20) for 10 min each, the cells were incubated with secondary antibodies in the dark for 1 h at room temperature. After the samples were washed three times with PBST for 10 min each, the cell nuclei were stained with 1 μg/mL DAPI (GoodBiotech, Wuhan, China) for 10 min at room temperature. For the mitochondrial-associated membrane (MAM) marker, fatty acid CoA ligase 4 (FACL4) mouse monoclonal antibody (Beyotime, Shanghai, China) was used for the colocalization. Fluorescence signals were observed under a Zeiss LSM 880 confocal microscope (Carl Zeiss, Oberkochen, Germany).

### 2.17 Statistical analysis

Data were analyzed by Student’s *t*-test for comparison between two groups, and one-way ANOVA with Tukey’s multiple comparison test for comparison among multiple groups. Statistical calculations were performed using Data Processing System (DPS) software (Tang and Zhang, 2013). Figures were visualized by GraphPad Prism 5.0 software (GraphPad) and generated by AI Illustrator (Adobe).

## 3 Results

### 3.1 Conserved protein sequences of CLIC in insects

**FIGURE 1:**
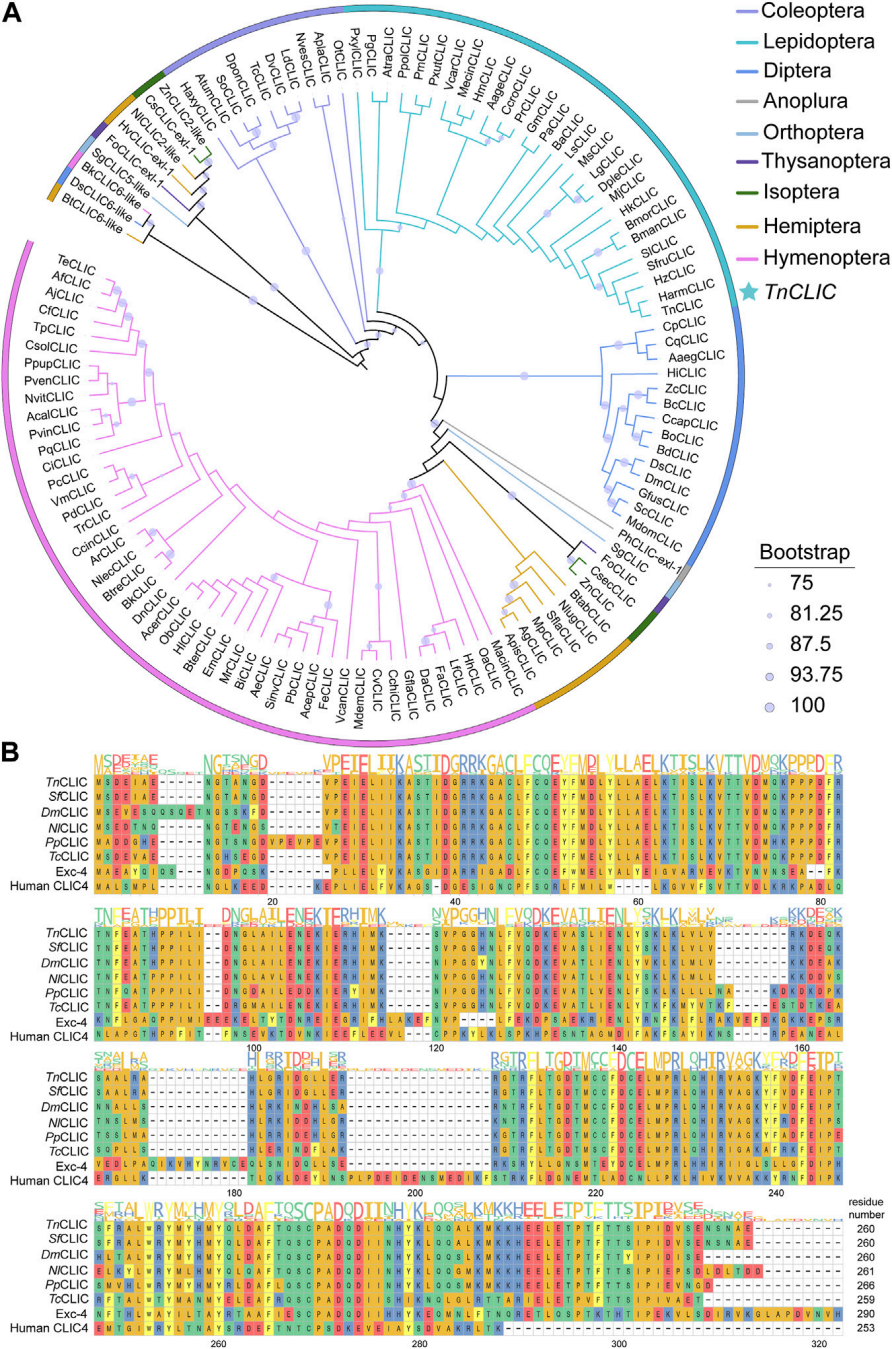
Phylogenetic analysis and multiple sequence alignment of chloride intracellular channel (CLIC) proteins from different species. **(A)** The maximum likelihood phylogenetic tree with bootstrapping at 1, 000 replicates using IQ-Tree software. Different colors represent different species according to the color code to the top right. Numbers of insect *CLIC* are listed in Supplementary Table S2, and the newly designated gene names of selected species are represented by a two/four-letter species abbreviation. *TnCLIC* is marked with a star. **(B)** A multi-sequence alignment of *CLIC* along with the consensus sequence logo from *Trichoplusia ni*, *Spodoptera frugiperda*, *Drosophila melanogaster*, *Nilaparvata lugens*, *Pteromalus puparum*, *Tribolium castaneum*, *Caenorhabditis elegans*, and *Homo sapiens*. Colours display distinct amino acid properties and height of the letters indicates conservation of amino acids.

The *Trichoplusia ni chloride intracellular channel* (*TnCLIC*) cDNA has a 783-bp ORF, encoding 260 amino acids, with a predicted molecular mass of 29.76 kDa and an isoelectric point of 5.66. Using the protein as a seed, we annotated *CLIC* in 110 insect species. The CLIC protein sequences selected for phylogenetic analysis are shown in the Supplementary material. Phylogenetic analysis results indicated that *TnCLIC* clustered with *HzCLIC*, *HaCLIC*, *SfCLIC*, and *SlCLIC* (Figure 1A). The corresponding species are *Helicoverpa zea*, *H. armigera*, *S. frugiperda* and *S. litura*. Among annotated genes, we found only one CLIC protein has been found in listed lepidopteran and coleopteran insects. No signal peptide was predicted for any insect CLIC protein sequence. Multiple sequence alignments (Figure 1B) showed that *Tn*CLIC is similar to CLIC of other insect species from different taxonomic orders, but not Exc-4 or human CLIC4. It is implicated that CLIC is a unique and evolutionarily conserved protein in most insects.

### 3.2 Cell death induction in Hi-5 cells after *TnCLIC* knockdown

**FIGURE 2:**
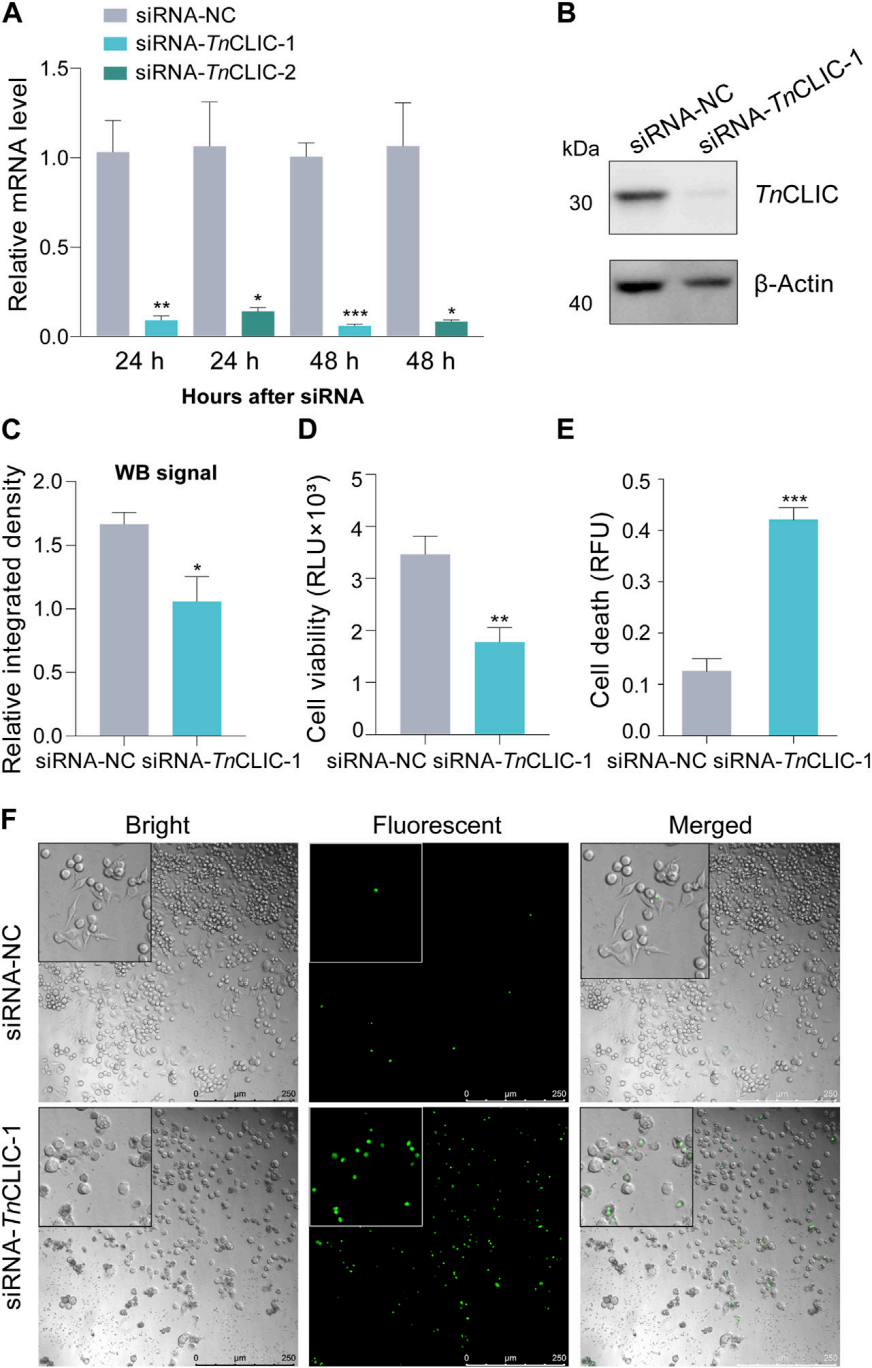
RNAi knockdown of the *TnCLIC* gene in Hi-5 cells. **(A)** RT‒qPCR of the mRNA level in Hi-5 cells transfected with negative control siRNA (siRNA-NC) or siRNA-1/2 targeting *TnCLIC* (siRNA-*Tn*CLIC-1/2, two pairs) for 24 and 48 h. Each treatment had three biological replicates. **(B)** Western blot of *Tn*CLIC protein levels at 48 h after treatment. β-actin was used as a loading control. **(C)** The relative integrated density for each band through the western blot signal (*n* = 3). **(D)** Viability of Hi-5 cells after treatment with siRNA-NC or siRNA-*Tn*CLIC-1 for 48 h (*n* = 4). RLU: relative luminescence unit. **(E)** Cell death of Hi-5 cells after incubation with siRNA-NC or siRNA-*Tn*CLIC-1 for 96 h (*n* = 3). RFU: relative fluorescence unit. **(F)** Merged (bright and fluorescent) images of Hi-5 cells transfected with siRNA-NC or siRNA-*Tn*CLIC-1 for 96 h. Bar = 250 µm. *p*-value of Student’s *t*-test: *p* < 0.05 (*), *p* < 0.01 (**) and *p* < 0.001 (***). Values are the mean ± SEM.

To explore the function, we performed *TnCLIC* knockdown experiments in Hi-5 cells *in vivo*. The efficiency of siRNA silencing was assessed by RT‒qPCR using two different siRNA pairs (Figure 2A; Student’s *t*-test for 24 h and 48 h: *t* = 5.2718, *df* = 4, *p* = 0.0062; *t* = 3.7030, *df* = 4, *p* = 0.0208; *t* = 12.1935, *df* = 4, *p* = 0.0003; *t* = 4.0650, *df* = 4, *p* = 0.0153). The results showed that RNAi significantly downregulated *TnCLIC* expression at 24 and 48 h by siRNA-*Tn*CLIC-1 compared with siRNA-*Tn*CLIC-2. Thus, the protein level of *TnCLIC* knockdown at 48 h was evaluated using siRNA-*Tn*CLIC-1 and the effect was validated by western blotting (Figures 2B, C; Student’s *t*-test for 48 h: *t* = 2.8087, *df* = 4, *p* = 0.0484). Correspondingly, luminescent and fluorescent values showed that siRNA-*Tn*CLIC-1 inhibited cell proliferation at 48 h (Figure 2D; *t* = 3.7808, *df* = 6, *p* = 0.0092) and induced cell death in Hi-5 cells at 96 h (Figure 2E; *t* = 8.6923, *df* = 4, *p* = 0.0010) compared with the negative control. Bright and fluorescent images clearly proved that siRNA-*Tn*CLIC-1 had lethal effects on the Hi-5 cell line at 96 h (Figure 2F). Meanwhile, Hi-5 cells transfected with siRNA-*Tn*CLIC-1 displayed changes in the morphology, showing that treated cells formed significantly fewer colonies and changed from spindle-shaped to rounded and shrinkable. Together, these results indicate that *Tn*CLIC is a protein essential for Hi-5 cell line survival.

### 3.3 Verification of cell death in Sf9 and S2 cells after *CLIC* knockdown

**FIGURE 3:**
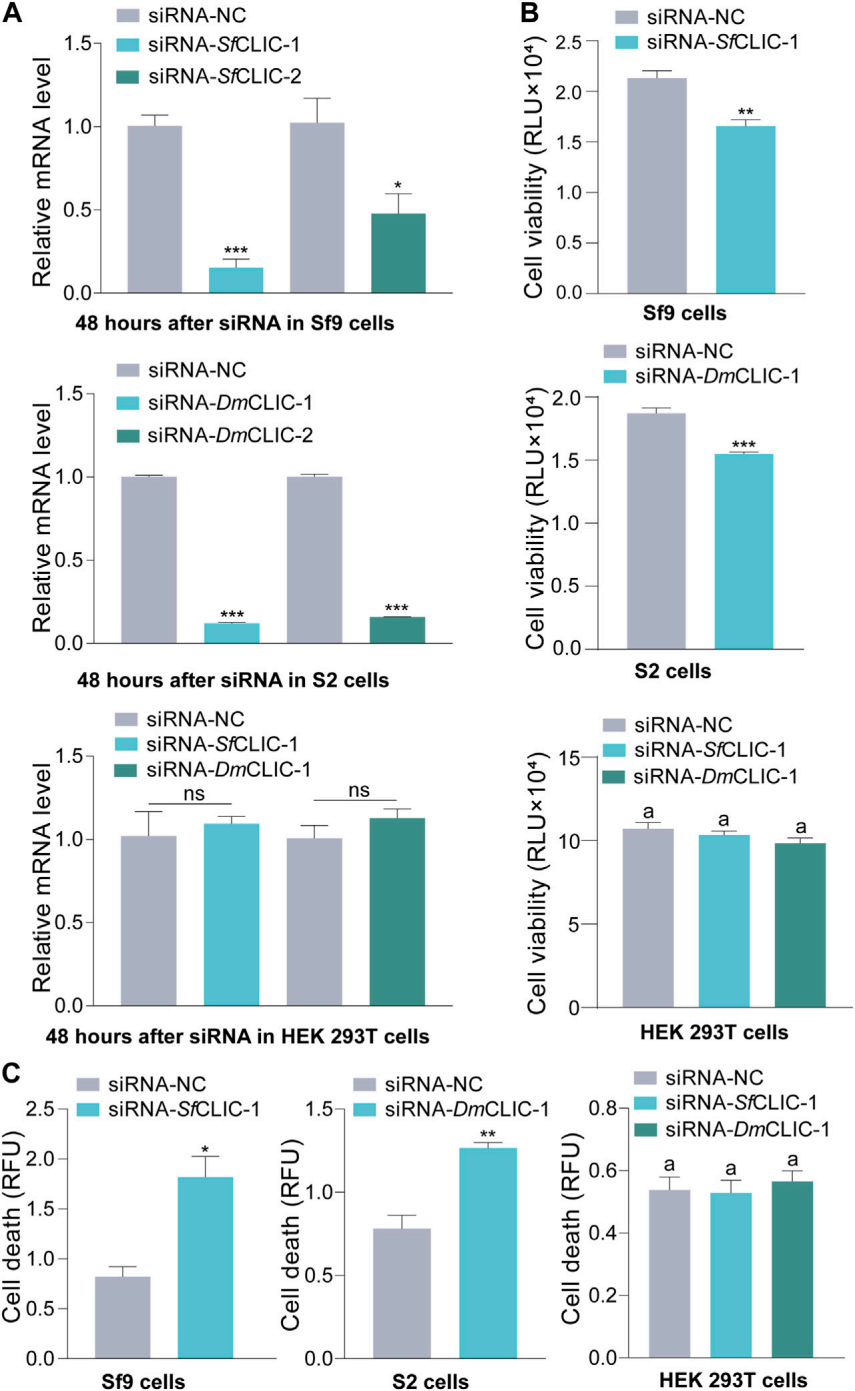
RNAi knockdown of the *CLIC* gene in Sf9 and S2 cells. **(A)** RT‒qPCR of the mRNA level of *SfCLIC* and *DmCLIC* after transfection with siRNA-*Sf*CLIC-1/2 and siRNA-*Dm*CLIC-1/2 for 48 h, respectively. siRNA-*Sf*CLIC-1 and siRNA-*Dm*CLIC-1 were transfected into HEK 293T cells to test the relative mRNA level of human *CLIC4*. Three biological replicates per sample were performed. **(B)** Cell viability after treatment with siRNA-*Sf*CLIC-1 or siRNA-*Dm*CLIC-1 for 48 h (*n* = 4). **(C)** Cell death after incubation with siRNA- *Sf*CLIC-1 or siRNA-*Dm*CLIC-1 for 48 h (*n* = 3). *p*-value of Student’s *t*-test: *p* < 0.05 (*), *p* < 0.01 (**), *p* < 0.001 (***) and ns means no significant difference. One-way ANOVA with Tukey’s multiple-comparison test at *p* < 0.05 was used in **(B,C)**. Values are the mean ± SEM.

To verify the lethal effects, *CLIC* knockdown experiments were performed in Sf9 and S2 cells *in vivo*. These siRNAs target *SfCLIC* or *DmCLIC*. Furthermore, these siRNAs were also respectively transfected into HEK 293T cells to verify siRNAs targeting insect *CLIC* genes had no significant effect on the human *CLIC4* gene in HEK 293T cells. The interference efficiency was detected by RT‒qPCR at 48 h post-transfection using two pairs of siRNA (Figure 3A; Student’s *t*-test for 48 h: *t* = 10.1865, *df* = 4, *p* = 0.0005; *t* = 2.8983, *df* = 4, *p* = 0.0442; *t* = 72.5871, *df* = 4, *p* < 0.0001; *t* = 49.9480, *df* = 4, *p* < 0.0001; *t* = 0.4805, *df* = 4, *p* = 0.6560; *t* = 1.2606, *df* = 4, *p* = 0.2760). The results demonstrated the consequential outcome of reduced *SfCLIC* and *DmCLIC* expression in Sf9 and S2 cells, and siRNAs that target insect *CLIC* did not affect *H. sapiens CLIC4* (*HsCLIC4*) expression since insect CLIC proteins are different from human CLIC4 by multiple sequence alignments. Besides a better interference efficiency, siRNA-1 targets similar sequence fragments for *TnCLIC*, *SfCLIC* and *DmCLIC*. Thus, siRNA-1 was used for follow-up experiments. Correspondingly, the protein level of *CLIC* knockdown was validated by western blotting (Supplementary Figure S2). In addition, luminescence and fluorescence signals showed that siRNA-1 inhibited cell proliferation (Figure 3B; *t* = 4.8206, *df* = 6, *p* = 0.0029; *t* = 6.9244, *df* = 6, *p* = 0.0004) and induced cell death at 48 h (Figure 3C; *t* = 4.3141, *df* = 4, *p* = 0.0125; *t* = 5.5414, *df* = 4, *p* = 0.0052) in Sf9 and S2 cells, rather than in the HEK 293T cell line (Figures 3B, C; ANOVA: *F*_(2, 9)_ = 1.8694, *p* = 0.2094; *F*_(2, 6)_ = 0.2356, *p* = 0.7971). It suggests that siRNAs that target insect *CLIC* only significantly induce cell death in insect cells but not in HEK 293T cells. This also indicates that siRNA-1 targeting *SfCLIC* or *DmCLIC* has no off-target effects on HEK 293T cells. In brief, these findings indicate that CLIC is vital for the survival of Sf9 and S2 insect cell lines.

### 3.4 Differential gene expression and functional enrichment analysis

**FIGURE 4:**
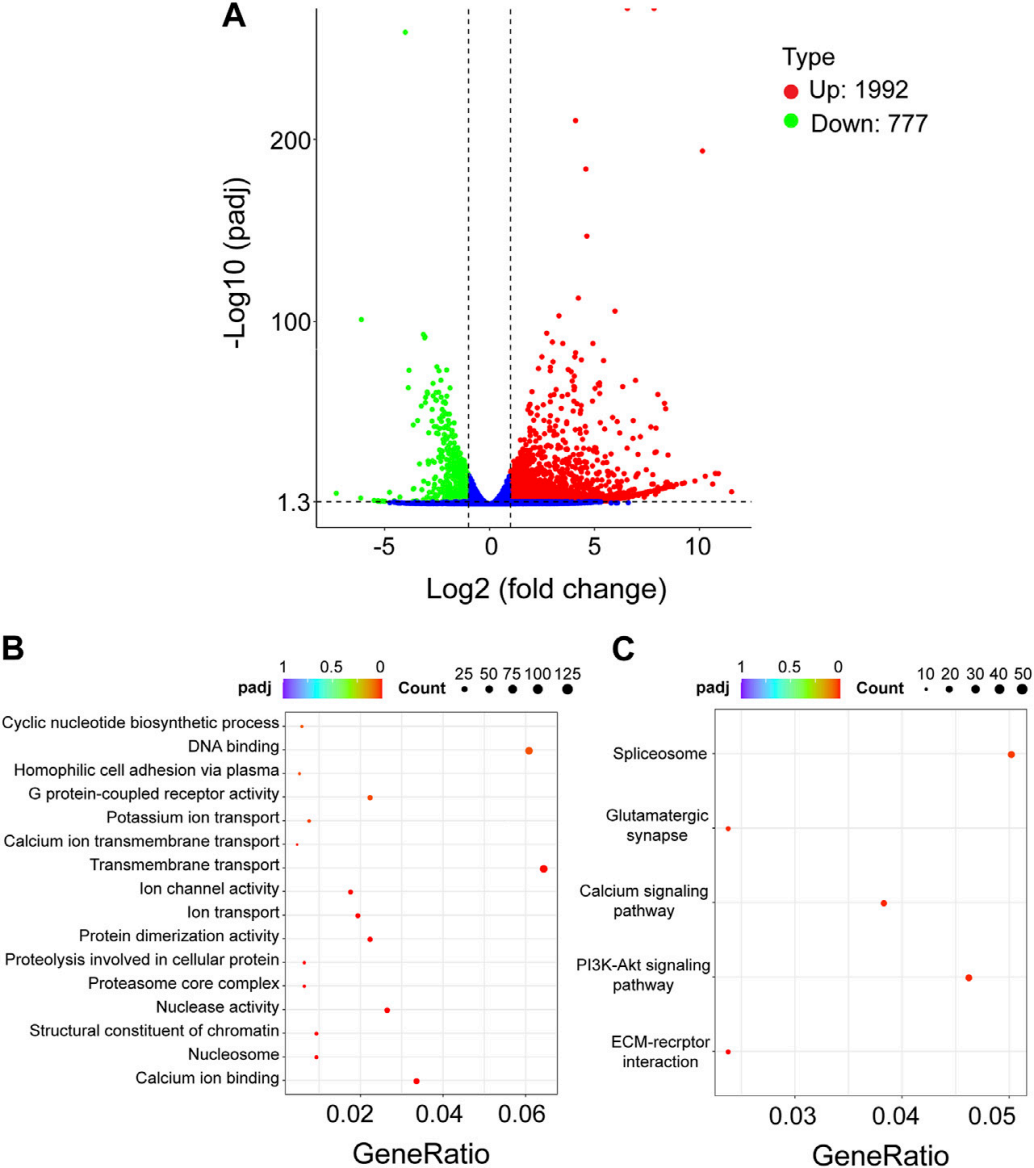
Transcriptomic analysis of Hi-5 cells treated with siRNA-NC vs. siRNA-*Tn*CLIC-1. **(A)** Volcano plot of differentially expressed genes (DEGs). Up: upregulated genes. Down: downregulated genes. **(B)** Significant statistics of Gene Ontology (GO) term enrichment. **(C)** Significant statistics of Kyoto Encyclopedia of Genes and Genomes (KEGG) pathway enrichment. Count: the number of DEGs in the term or pathway, the larger dot represents more DEGs number; GeneRatio: the number of DEGs/the total number of genes in this pathway; padj: *p* < 0.05 after Bonferroni correction, redder color represents greater significance.

**FIGURE 5:**
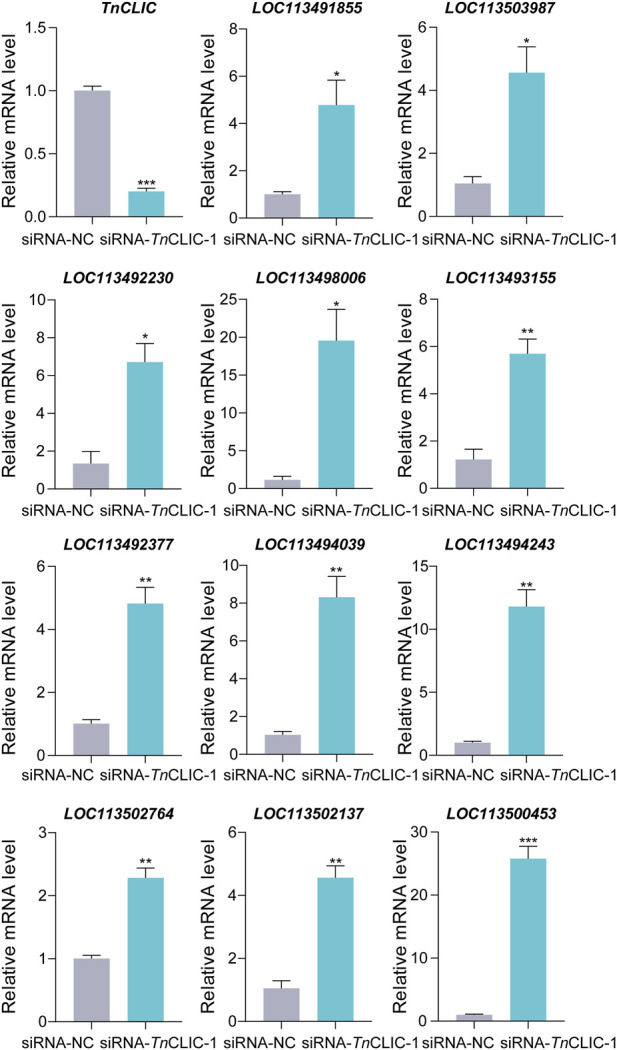
RT‒qPCR analysis of the relative mRNA level of candidate genes involved in Ca^2+^ signaling or encoding Ca^2+^ channels after *TnCLIC* knockdown. Values are the mean ± SEM. The experiment had three biological replicates. *p*-value of Student’s *t*-test: *p* < 0.05 (*), *p* < 0.01 (**) and *p* < 0.001 (***). LOC113491855: voltage-dependent T-type calcium channel subunit α-1G-like; LOC113503987: 1-phosphatidylinositol 4, 5-bisphosphate phosphodiesterase; LOC113492230: troponin C; LOC113498006: muscle calcium channel subunit α-1-like; LOC113493155: calcium/calmodulin-dependent 3′, 5′-cyclic nucleotide phosphodiesterase 1; LOC113492377: caltractin-like; LOC113494039: frequenin-1; LOC113494243: calbindin-32; LOC113502764: calcium release-activated calcium channel protein 1-like; LOC113502137: calmodulin-A-like; LOC113500453: tyrosine kinase receptor Cad96Ca.

To further identify the genes and pathways involved in the cell death phenotype of Hi-5 cells treated with siRNA-*Tn*CLIC-1, the transcriptome profiles of siRNA-NC and siRNA-*Tn*CLIC-1 lines were sequenced, compared and analyzed, referencing the Hi-5 cell line genome. Raw data for the transcriptome were deposited at NCBI sequence read archive (SRA) under BioProject PRJNA922752. One dataset of differentially expressed genes (DEGs) was generated as a result of the knockdown of *TnCLIC*: siRNA-NC vs. siRNA-*Tn*CLIC-1. The DEGs affected by siRNA-*Tn*CLIC-1 resulted in 1992 upregulated DEGs and 777 downregulated DEGs (Figure 4A). Then, Gene Ontology (GO: biological process, molecular function, and cellular component) and Kyoto Encyclopedia of Genes and Genomes (KEGG) pathway analyses were performed. The results showed that the GO terms were significantly enriched in DNA binding, calcium ion binding and transmembrane transport (Figure 4B). The KEGG pathway analysis showed that the spliceosome, PI3K-Akt and calcium signaling pathways were significantly enriched for siRNA-NC vs. siRNA-*Tn*CLIC-1 (Figure 4C). After GO and KEGG analyses, “calcium ion binding” and “calcium signaling” related terms and pathways were significantly co-enriched. To verify the relevant RNA-seq data, RT‒qPCR was performed and 11 candidate genes involved in Ca^2+^ signaling or encoding Ca^2+^ channels were significantly upregulated after *TnCLIC* knockdown (Figure 5; Student’s *t*-test in order: *t* = 18.2152, *df* = 4, *p* = 0.0001; *t* = 3.5751, *df* = 4, *p* = 0.0233; *t* = 4.1512, *df* = 4, *p* = 0.0142; *t* = 4.5661, *df* = 4, *p* = 0.0103; *t* = 4.4371, *df* = 4, *p* = 0.0114; *t* = 5.9185, *df* = 4, *p* = 0.0041; *t* = 7.2197, *df* = 4, *p* = 0.0020; *t* = 6.5403, *df* = 4, *p* = 0.0028; *t* = 8.0923, *df* = 4, *p* = 0.0013; *t* = 7.9005, *df* = 4, *p* = 0.0014; *t* = 7.9348, *df* = 4, *p* = 0.0014; *t* = 12.6456, *df* = 4, *p* = 0.0002). Thus, these results proved that *TnCLIC* widely participates in diverse pathways in response to knockdown, especially involving the levels of intracellular calcium ions.

### 3.5 Cell death and increased intracellular calcium ions after *TnCLIC* knockdown

**FIGURE 6:**
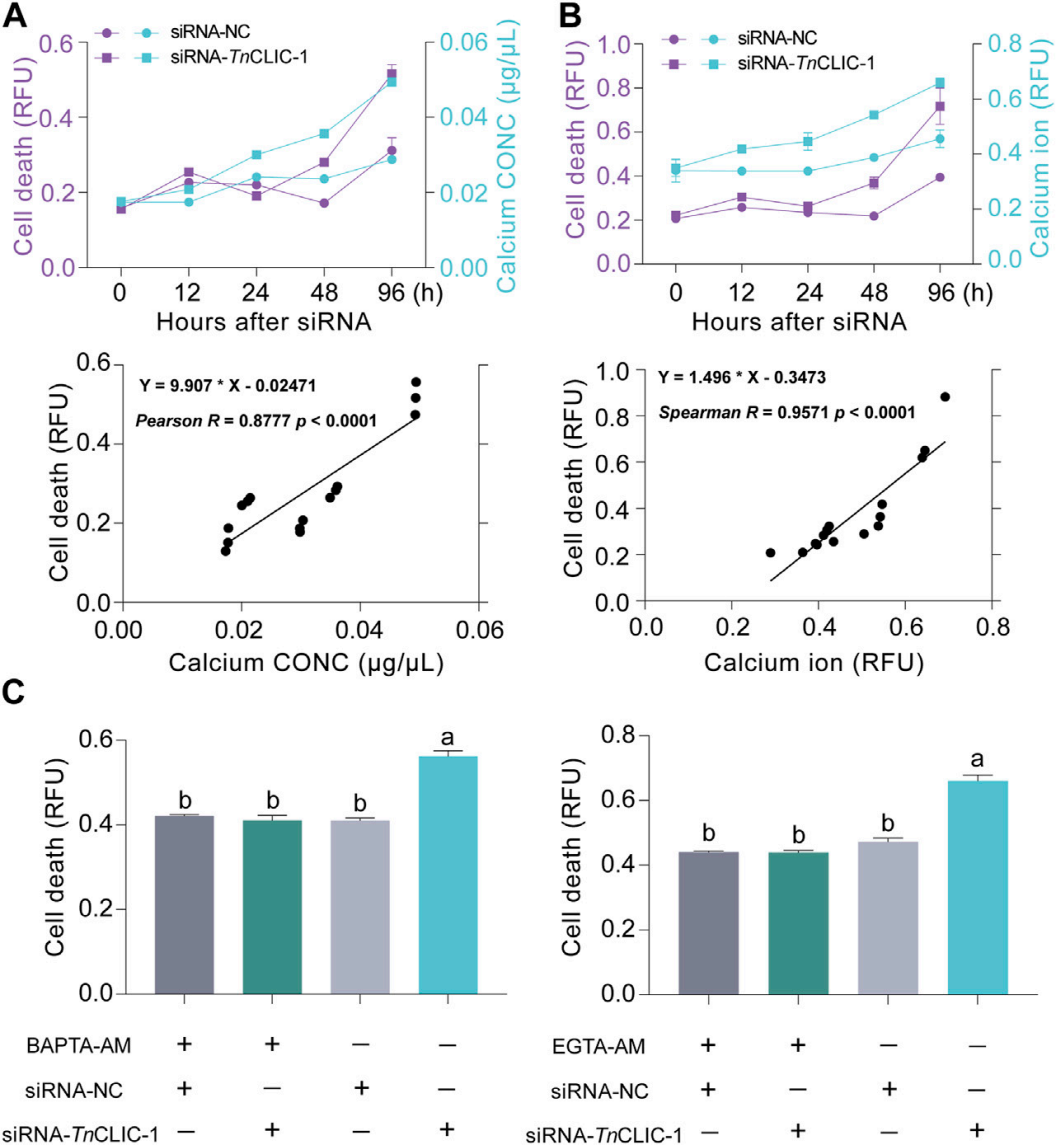
The correlation between calcium ion increase and cell death after *TnCLIC* knockdown. **(A)** Dynamic changes and correlation analysis of cell death and calcium concentration (*n* = 3). CONC: concentration. **(B)** Dynamic changes and correlation analysis of cell death and intracellular calcium ion using fluorescent probes (*n* = 3). **(C)** Cell death was evaluated in *TnCLIC* mutant cells after the treatment of Ca^2+^ chelators BAPTA-AM or EGTA-AM (*n* = 4). One-way ANOVA with Tukey’s multiple-comparison test at *p* < 0.05 was used. Values are the mean ± SEM.

To validate the results of transcriptomic analysis, the calcium concentration and cell death were detected using siRNA-*Tn*CLIC-1 treatment for 0, 12, 24, 48, and 96 h against Hi-5 cells. Intracellular calcium concentration was detected by colorimetric and fluorescent methods, respectively. The results showed that the intracellular calcium concentration was significantly increased compared with that in the control (Figures 6A, B; Student’s *t*-test for 0 h: *t* = 1.6646, *df* = 4, *p* = 0.1713; *t* = 0.1652, *df* = 4, *p* = 0.8768. Student’s *t*-test for 12 h: *t* = 8.1095, *df* = 4, *p* = 0.0013; *t* = 19.9624, *df* = 4, *p* < 0.0001. Student’s *t*-test for 24 h: *t* = 27.2958, *df* = 4, *p* < 0.0001; *t* = 3.3588, *df* = 4, *p* = 0.0283. Student’s *t*-test for 48 h: *t* = 29.7562, *df* = 4, *p* < 0.0001; *t* = 21.8847, *df* = 4, *p* < 0.0001. Student’s *t*-test for 96 h: *t* = 307.9769, *df* = 4, *p* < 0.0001; *t* = 5.6127, *df* = 4, *p* = 0.0050). Correspondingly, cell death significantly increased (Figures 6A, B; Student’s *t*-test for 0 h: *t* = 0.1600, *df* = 4, *p* = 0.8806; *t* = 0.8375, *df* = 4, *p* = 0.4494. Student’s *t*-test for 12 h: *t* = 2.8804, *df* = 4, *p* = 0.0450; *t* = 3.5508, *df* = 4, *p* = 0.0238. Student’s *t*-test for 24 h: *t* = 3.2225, *df* = 4, *p* = 0.0322; *t* = 1.9195, *df* = 4, *p* = 0.1273. Student’s *t*-test for 48 h: *t* = 7.5309, *df* = 4, *p* = 0.0017; *t* = 5.2971, *df* = 4, *p* = 0.0061. Student’s *t*-test for 96 h: *t* = 4.9047, *df* = 4, *p* = 0.0080; *t* = 3.8863, *df* = 4, *p* = 0.0177). Then, correlation analyses of cell death and intracellular calcium ion were performed. Pearson and Spearman’s correlation analysis results show that intracellular calcium ion has a significant positive correlation with cell death (*R* = 0.8777, *p* < 0.0001; *R* = 0.9571, *p* < 0.0001). To further investigate the effect of Ca^2+^ in the cell death of *TnCLIC* mutant cells, Hi-5 cells were pretreated with Ca^2+^ chelators BAPTA-AM or EGTA-AM. After the treatment, cell death decreased significantly even after incubation with siRNA-*Tn*CLIC-1 for 96 h (Figure 6C; ANOVA: *F*_(3, 12)_ = 61.1184, *p* < 0.0001; *F*_(3, 12)_ = 84.7996, *p* < 0.0001). In general, these findings indicate that cell death is involved in level increases of intracellular calcium ions after *TnCLIC* knockdown.

### 3.6 Cell death pattern and immunofluorescence localization of *Tn*CLIC

**FIGURE 7:**
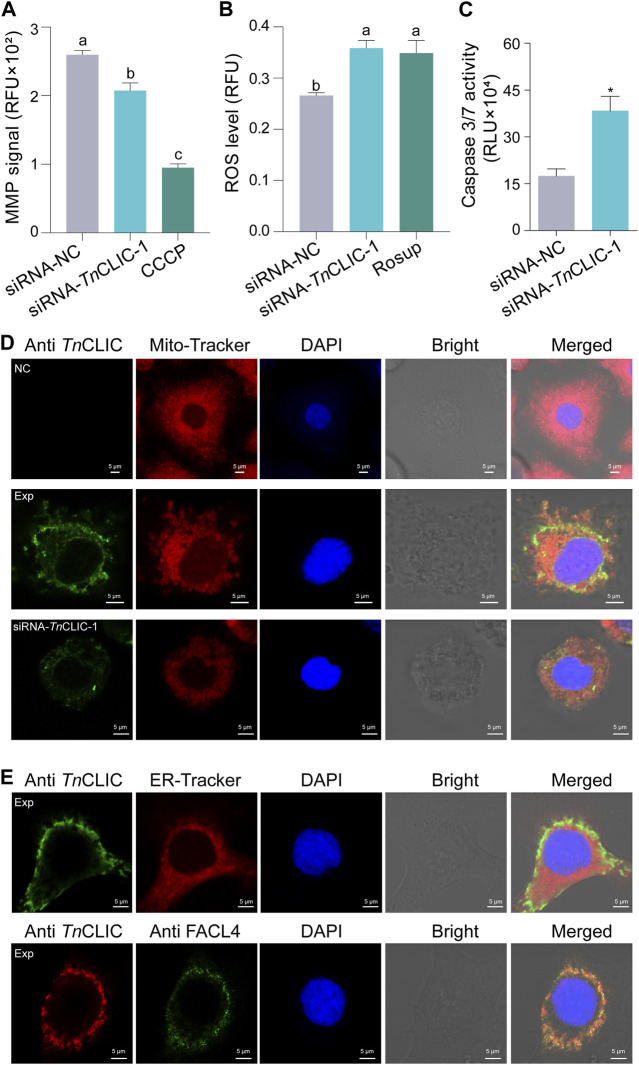
Lethal pattern analysis mediated *TnCLIC* knockdown and *Tn*CLIC sublocalization. **(A)** Mitochondrial membrane potential (MMP). CCCP: carbonyl cyanide 3-chlorophenylhydrazone. **(B)** Reactive oxygen species (ROS) level. Rosup: a ROS-positive inducer. One-way ANOVA with Tukey’s multiple-comparison test at *p* < 0.05 was used and four biological replicates were evaluated in **(A,B)**. **(C)** Caspase 3/7 activity (*n* = 3). *p*-value of Student’s *t*-test: *p* < 0.05 (*). Values are the mean ± SEM. Experimental treatments in **(A–C)** were applied to Hi-5 cells after incubation with siRNA-NC or siRNA-*Tn*CLIC-1 for 96 h. **(D)** Immunofluorescence staining of *Tn*CLIC in Hi-5 cells. NC, negative control, means non-transfected Hi-5 cells without the primary antibody. Exp means non-transfected Hi-5 cells with the primary antibody. siRNA-*Tn*CLIC-1 means Hi-5 cells transfected with siRNA-*Tn*CLIC-1 and incubated with the primary antibody. **(E)** *Tn*CLIC sublocalization in the endoplasmic reticulum and colocalization with fatty acid CoA ligase 4 (FACL4). Exp means Hi-5 cells with the primary antibody. The corresponding negative control group in Supplementary Figure S4. Nuclei were stained with DAPI (blue). Bar = 5 µm.

Various cellular signals, including the intracellular mitochondrial membrane potential (MMP), reactive oxygen species (ROS) and caspase 3/7 activity, were detected to uncover the cell death pattern. The results showed that intracellular MMP declined, and the ROS level and caspase 3/7 activity were increased when siRNA-*Tn*CLIC-1 treatment was applied for 96 h (Figures 7A–C; ANOVA: *F*_(2, 9)_ = 107.8509, *p* < 0.0001; *F*_(2, 9)_ = 9.0822, *p* = 0.0069; Student’s *t*-test: *t* = 3.9841, *df* = 4, *p* = 0.0163). In addition, immunofluorescence images revealed that the *Tn*CLIC protein located in the cytoplasm is mainly distributed in the mitochondria (Figure 7D) and partly distributed in the endoplasmic reticulum (Figure 7E; Supplementary Figure S4). Meanwhile, fatty acid CoA ligase 4 (FACL4), a marker enriched in the mitochondrial-associated membrane (MAM) fraction colocalizes with *Tn*CLIC protein in Hi-5 cells.

## 4 Discussion

As mentioned earlier, vertebrate chloride intracellular channels (CLICs) are highly conserved proteins in the soluble form (Argenzio and Moolenaar, 2016). Besides, the bacterial stringent starvation protein SspA and the yeast prion protein Ure2p also share structural homology with CLICs (Oakley, 2005; Littler et al., 2010; Gururaja Rao et al., 2017). In the present study, CLIC protein was been annotated in most insects and other members of the *CLICs-like* genes family are present in a few insect species, including *Bemisia tabaci*, *Nilaparvata lugens*, *Belonocnema kinseyi*, *Drosophila subobscura*, *Schistocerca gregaria*, *Zootermopsis nevadensis*, *Cryptotermes secundus* and *Frankliniella occidentalis*. Multiple sequence alignments suggest that selected insect CLIC proteins are conserved, consisting of approximately 260 amino acids. In addition, the *Tn*CLIC protein can be expressed by the prokaryotic system, which proves the protein solubility. These results are consistent with those reported for the fundamental biochemical properties of CLICs in the literature (Howell et al., 1996; Duncan et al., 1997). To explain the differences in CLICs between vertebrates and invertebrates, insect CLIC may be a good model to compare the protein structure and molecular functions.

For a long time, cell death has been a fascinating mystery and is largely divided into programmed cell death (PCD) and necrosis (Degterev and Yuan, 2008). Cell apoptosis is the most widely studied type of PCD and is an essential process regulated by the classical caspase pathway (Van Opdenbosch and Lamkanfi, 2019). To date, more reports and cases have uncovered the apoptosis mechanism of mammalian CLIC4 in different cancer cells (Xu et al., 2013; Yokoyama et al., 2021). For example, knocking down *CLIC4* enhanced ATP-induced apoptosis in head and neck squamous carcinoma cell lines (Xue et al., 2016). Here, decreased cell viability and increased cell death after *TnCLIC* knockdown in Hi-5 cells show that insect CLIC is important for cell survival. The phosphatidylinositol-3 kinase (PI3K)-Akt signaling pathway, which is related to apoptosis, was highlighted by KEGG analysis. Moreover, the activity of caspase 3/7 was enhanced after transfection with siRNA-*Tn*CLIC-1. These findings in Hi-5 cells are consistent with earlier observations in mammalian cells and imply that knocking down insect *CLIC* may be associated with cell apoptosis. Nevertheless, additional apoptotic experiments are needed to prove this hypothesis.

Calcium ions are crucial mediators of versatile cellular activities (Berridge et al., 1998), including cell proliferation and apoptosis (Orrenius et al., 2003). In living cells, cellular calcium transport is not controlled by a single ion channel (Armstrong and Cota, 1991). A previous study demonstrated that mammalian CLIC4 modulates mitochondrial calcium homeostasis under physiological and pathological conditions (Ponnalagu et al., 2022). In our current research, the enrichment of the GO terms “transmembrane transport,” “ion transport,” and “ion channel activity” indicates that the basic function of insect CLIC as an ion channel was changed after RNAi. Meanwhile, we can speculate that potassium and calcium ions may be involved in the CLIC physiological function according to the GO and KEGG statistical enrichment analyses of “potassium ion transport,” “calcium ion transmembrane transport,” “calcium ion binding” and “calcium signaling pathway,” respectively. Afterward, 11 candidate genes involved in Ca^2+^ signaling or encoding Ca^2+^ channels by RT‒qPCR and the experimental results of increased intracellular calcium levels in transfected Hi-5 cells directly prove the transcriptome data. This means that the increases in intracellular calcium ions is occurred after *TnCLIC* knockdown in Hi-5 cells.

Several groups of ion channels are distributed inside the membranes of intracellular organelles (Xu et al., 2015), including mitochondria (Ponnalagu et al., 2016b), Golgi apparatus (Almeida et al., 2020) and endoplasmic reticulum (ER) (Ponnalagu et al., 2016a). Since mitochondrial depolarization (Lu et al., 2018) and reactive oxidative stress (ROS) (Kiselyov and Muallem, 2016) play a highly relevant role in apoptosis by modulating of mammalian CLICs, mitochondrial membrane potential (MMP) and ROS generation are thus commonly used to gain a better understanding of their function. For example, the accumulation of cellular ROS, as a signaling module, was increased by deleting *CLIC4* from murine 6DT1 breast tumor cells using CRISPR (Al Khamici et al., 2022). Similarly, the decreased intracellular MMP in the study after *TnCLIC* knockdown in Hi-5 cells suggests that *Tn*CLIC may play a pivotal role in the maintenance of mitochondrial function. In addition, the intimate connection between calcium ions and mitochondrial function (Bravo-Sagua et al., 2017) is corroborated by the increased intracellular calcium levels described above. For the localization of CLIC in insects, immunofluorescence images revealed that the *Tn*CLIC protein is mainly distributed in the mitochondria and colocalizes with fatty acid CoA ligase 4, a mitochondrial-associated membrane (MAM) marker (Ponnalagu et al., 2016b). Although CLIC1-6 are distributed in ER or mitochondria (Ponnalagu et al., 2016a; Rao et al., 2020), the current result does not completely rule out the possibility of other organelles for *Tn*CLIC. In addition, other MAM markers should be verified in the *Tn*CLIC protein colocalization to avoid false positive or unspecific results. In general, our results suggest that insect CLIC is likely to be important as a mediator of mitochondrial physiology for overall cell fitness.

Mammalian CLIC4 is an essential molecular component of cellular anion channels with poorly selective and redox-regulated activity (Proutski et al., 2002; Singh and Ashley, 2007). Since amphotericin B and rapamycin were screened as small molecule inhibitors to CLIC4 via a high-performance computing-powered blind-docking approach (Olotu et al., 2023), CLIC4 is expected to develop a novel target of clinical potential (Leanza et al., 2013). However, there are fewer applications for channel-targeting insecticides. In this work, we found evidence of cell death in the Hi-5, Sf9, and S2 cell lines after *CLIC* knockdown. However, the subject is not the cabbage looper *T. ni* after all. Additionally, sequence alignments showed that only one CLIC protein exists in listed lepidopteran insects with high conservation. Knocking down *CLIC* is likely to possess cell death-inducing activity in more insects, but verification of its live functionality should be further researched.

## 5 Conclusion

In general, we identified a *Tn*CLIC protein from the Hi-5 cell line originating from the cabbage looper *T. ni* and revealed the cell death phenotype after *TnCLIC* knockdown. Our results showed that cell death was accompanied by level increases of intracellular calcium ions in Hi-5 cells. Although it is not the only survival-related protein in Hi-5 cells, the broad-spectrum distribution of CLIC in most lepidopteran pests makes it a potential insecticide candidate target that should be considered in forthcoming studies. Future research should concentrate on the structure of insect CLIC and the search for small molecule compounds based on the key amino acid residues. In short, CLIC is conserved in most insects and vital for cell survival. Our research results on insect CLIC will lay the foundation for the study of the cell death mechanism.

## Data Availability

The datasets presented in this study can be found in online repositories. The names of the repository/repositories and accession number(s) can be found below: https://www.ncbi.nlm.nih.gov/, PRJNA922752.
