# Supplementary figures and images for: Chloride intracellular channel gene knockdown induces insect cell lines death and level increases of intracellular calcium ions
Source: Front Physiol. 2023 Jul 6;14:1217954. doi: 10.3389/fphys.2023.1217954 (PMC10356983; doi:10.3389/fphys.2023.1217954)

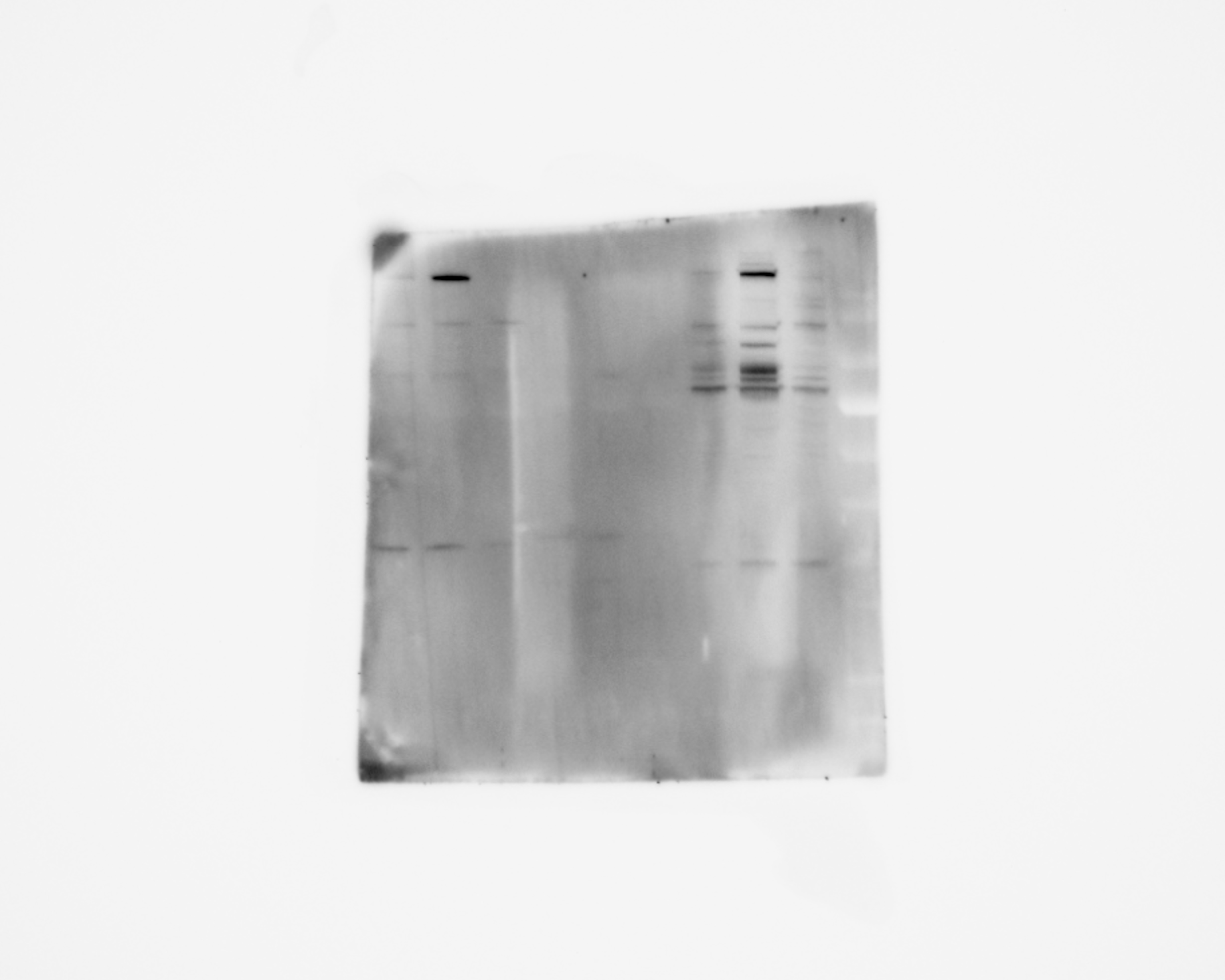

Supplement: Supplementary file 1 [file Image3.TIF]

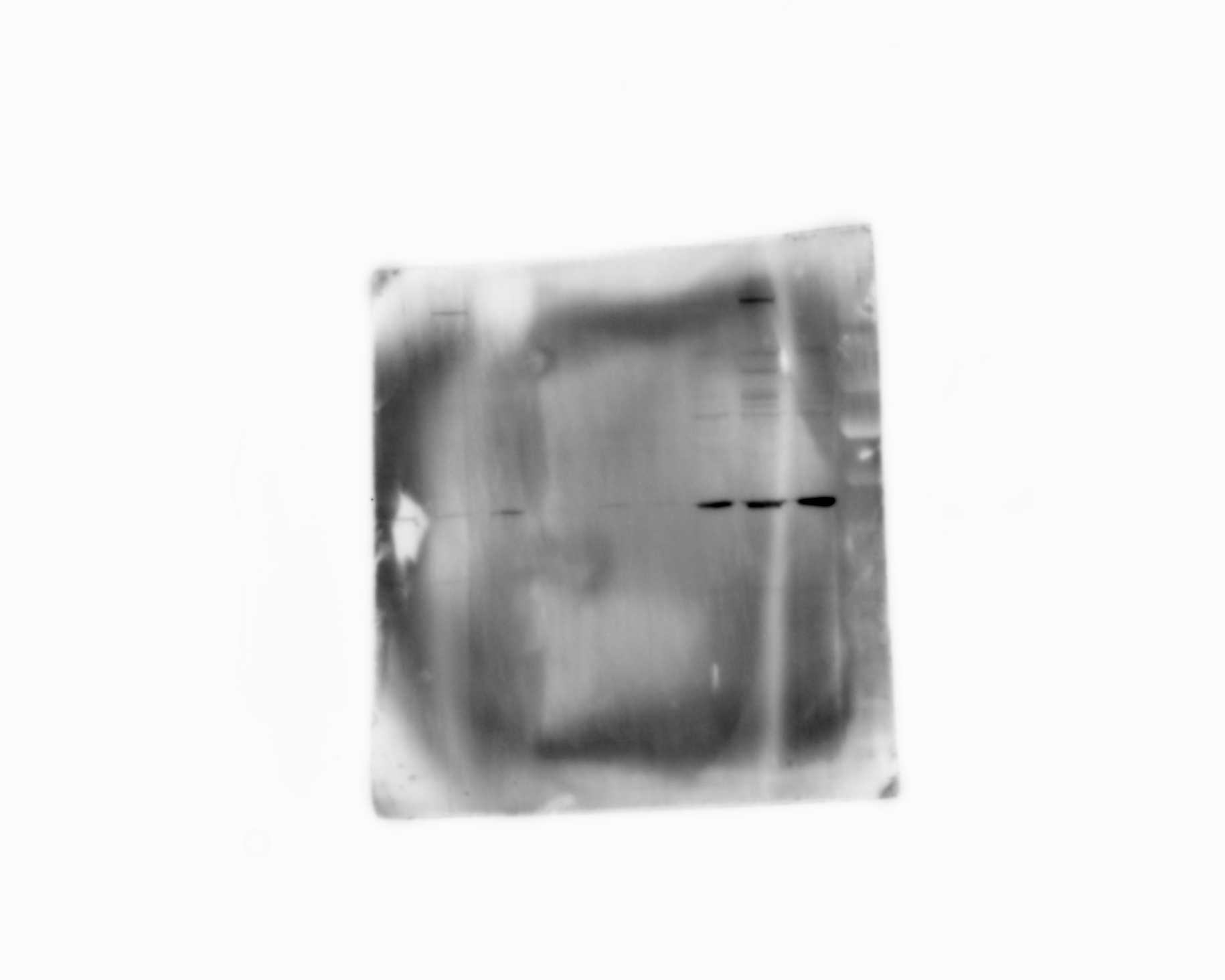

Supplement: Supplementary file 2 [file Image4.TIF]

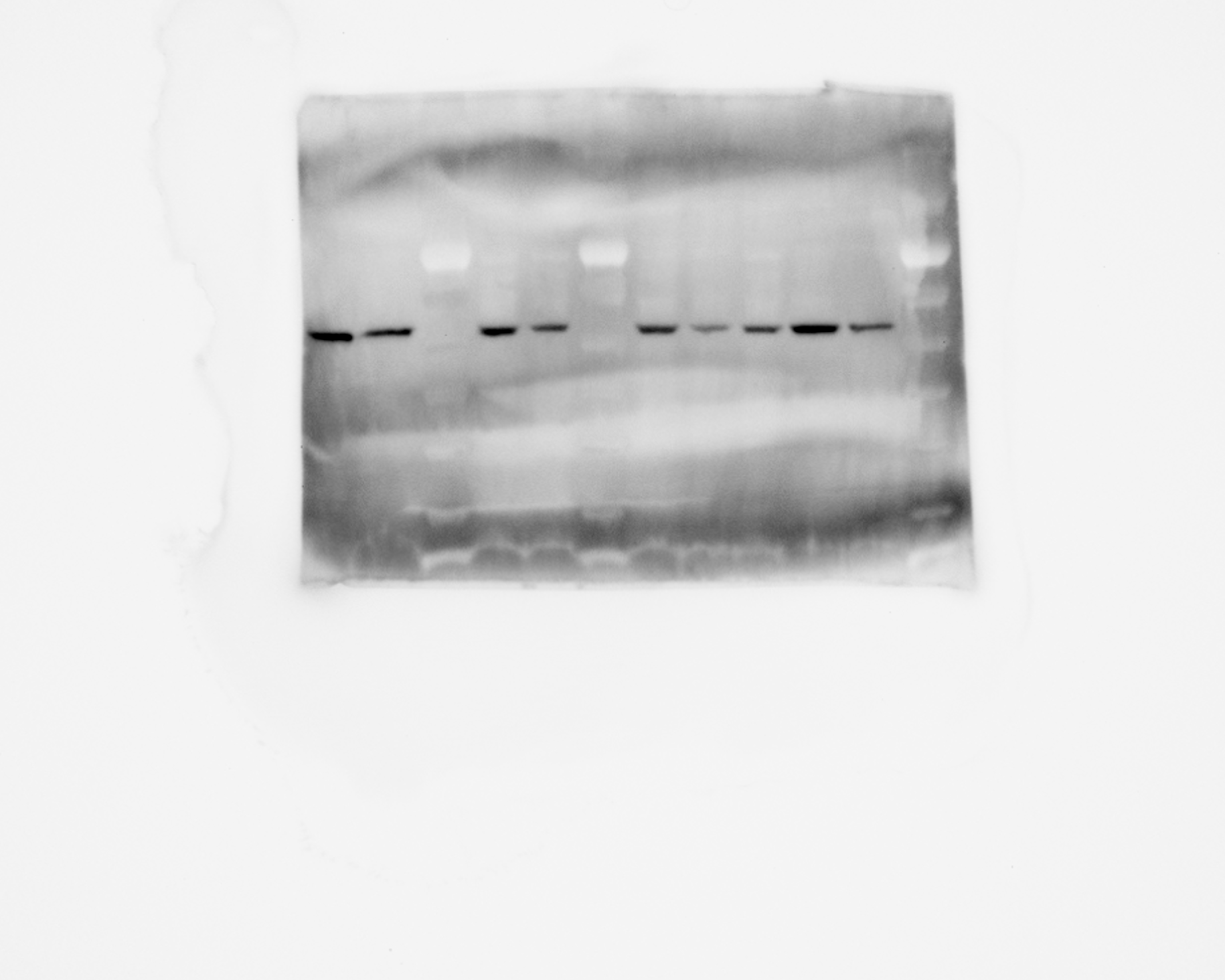

Supplement: Supplementary file 3 [file Image2.TIF]

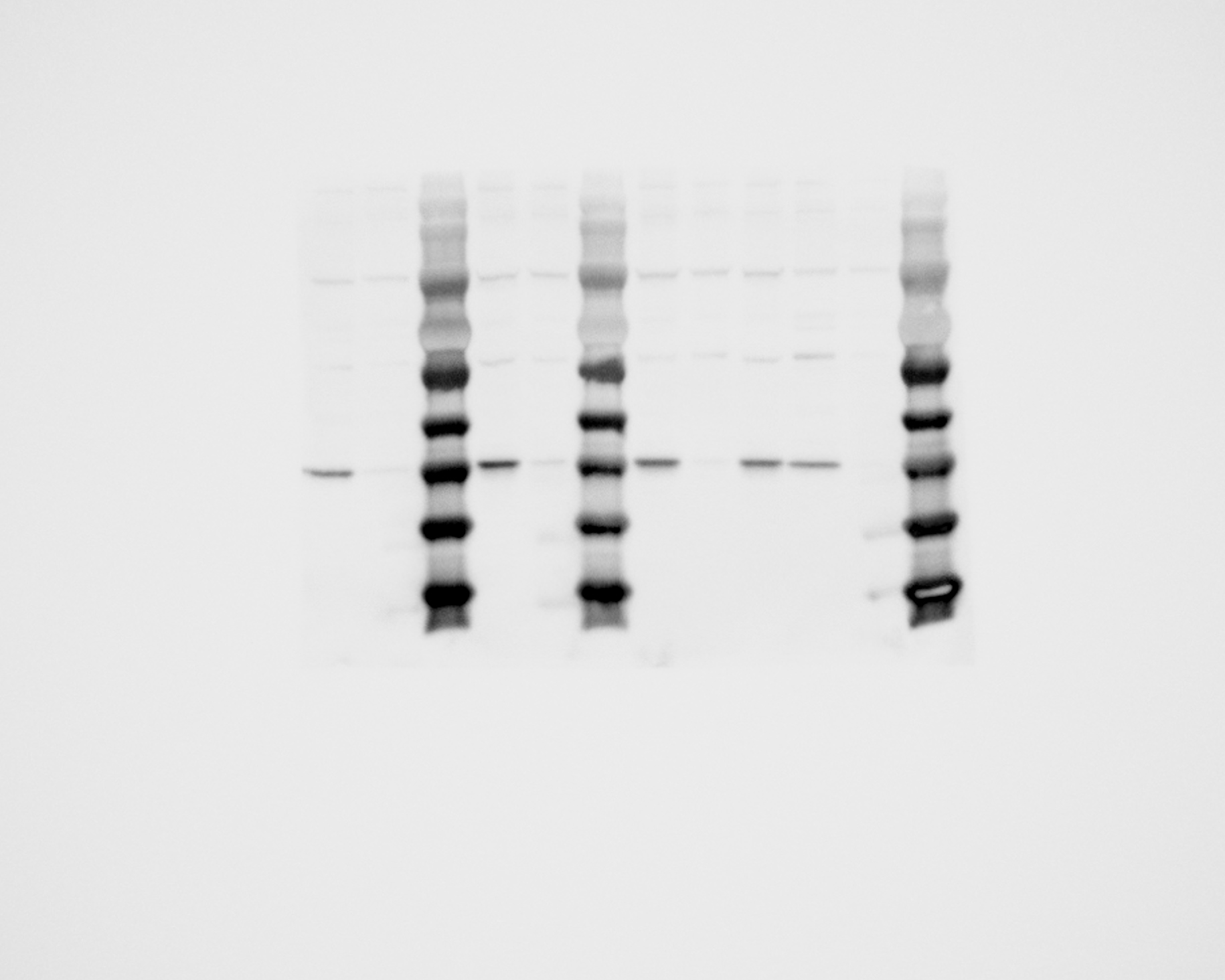

Supplement: Supplementary file 4 [file Image1.TIF]
