# Supplementary material for: Chloride intracellular channel gene knockdown induces insect cell lines death and level increases of intracellular calcium ions
Source: Front Physiol. 2023 Jul 6;14:1217954. doi: 10.3389/fphys.2023.1217954 (PMC10356983; doi:10.3389/fphys.2023.1217954)
Supplement: Supplementary file 5 [file DataSheet1.docx]

Supplementary Material

***Chloride intracellular channel* gene knockdown induces insect cell lines death** **and level increases of intracellular calcium ions**

Jiqiang Song, Yanping Yu, Zhichao Yan, Shan Xiao, Xianxin Zhao, Fang Wang, Qi Fang, Gongyin Ye^*^

*** Correspondence:** Gongyin Ye: chu@zju.edu.cn

# Supplementary Data

**CLICs amino acid sequences through gene annotation**

>*Ppup*CLIC

MADDGHENGTSNGDVPEVPEVPEIELIIKASTIDGRRKGACLFCQEYFMDLYLLAELKTISLKVTTVDMHKPPPDFRTNFQATPPPILIDNGDAILEDDKIERYIMKNIPGGHNLFVQDKEVATLVENLFSKLKLLLLNAKDKDKDPKTSSLMAHLRRIDEHLGRKGTRFLTGDTMCCFDCELMPRLQHIRVAGKYFADFEIPESMVHLWRYMYHMYRLDAFLQSCPADQDIINHYKLQQSMKMKKHEELETPTFTTSIPIEVNGD

>*Nvit*CLIC

MADDGHENGTSNGDVPEVPEVPEIELIIKASTIDGRRKGACLFCQEYFMDLYLLAELKTISLKVTTVDMHKPPPDFRTNFQATPPPILIDNGDAILEDDKIERYIMKNIPGGHNLFVQDKEVATLVENLFSKLKLLLLNAKDKDKDPKTSSLMAHLRRIDEHLGRKGTRFLTGDTMCCFDCELMPRLQHIRVAGKYFADFEIPESMVHLWRYMYHMYRLDAFLQSCPADQDIINHYKLQQSMKMKKHEELETPTFTTSIPIEVNGD

>*Acal*CLIC

MQRDDDDDDASTIDGRRKGACLFCQEYFMDLYLLAELKTISLKVTTVDMHKPPPDFRTNFEATPPPILIDNGNAILEDDKIERYIMKNIPGGHNLFVQDKEVATLVENLFSKLKLLLLNAKDKDKDPKTSSLMAHLRRIDEHLGRKGTRFLTGDTMCCFDCELMPRLQHIRVAGKYFADFEIPESMVHLWRYMYHMYRLDAFLQSCPADQDIINHYKLQQSMKMKKHEELETPTFTTSIPIEVNGD

>*Pvin*CLIC

MDGNAAVLNIQLGCSYKATASTIDGRRKGACLFCQEYFMDLYLLAELKTISLKVTTVDMHKPPPDFRTNFQATPPPILIDNGDAILEDDKIERYIMKNIPGGHNLFVQDKEVATLVENLFSKLKLLLLNAKDKDKDPKTSSLMAHLRRIDEHLGRKGTRFLTGDTMCCFDCELMPRLQHIRVAGKYFADFEIPESMVHLWRYMYHMYRLDAFLQSCPADQDIINHYKLQQSMKMKKHEELETPTFTTSIPIEVNGD

>*Pq*CLIC

MDLYLLAELKTISLKVTTVDMHKPPPDFRTNFQATPPPILIDNGDAILEDDKIERYIMKNIPGGHNLFVQDKEVATLVENLFSKLKLLLLNAKDKDKDPKTSSLMAHLRRIDEHLGRKGTRFLTGDTMCCFDCELMPRLQHIRVAGKYFADFEIPESMVHLWRYMYHMYRLDAFLQSCPADQDIINHYKLQQSMKMKKHEELETPTFTTSIPIEVNGD

>*Pven*CLIC

MADDGHENGTSNGDVPEVPEVPEIELIIKASTIDGRRKGACLFCQEYFMDLYLLAELKTISLKVTTVDMHKPPPDFRTNFQATPPPILIDNGDAILEDDKIERYIMKNIPGGHNLFVQDKEVATLVENLFSKLKLLLLNAKDKDKDPKTSSLMAHLRRIDEHLGRKGTRFLTGDTMCCFDCELMPRLQHIRVAGKYFADFEIPESMVHLWRYMYHMYRLDAFLQSCPADQDIINHYKLQQSMKMKKHEELETPTFTTSIPIEVNGD

>*Te*CLIC

MIPEKSEPTTQNVKIYFMIKYEVIDDLTKILTAKSFATNASTIDGRRKGACLFCQEYFMDLYLLAELKTISLKVTTVDMQKPPPDFRTNFQATPPPILIDNGDAILENEKIERHIMKNIPGGHNLFIQDKDVATLVENLFSKLKLLLLNAKDKDKDPKSSSLMAHLRKIDEHLGRKRTRFLTGDTMCCFDCELMPRLQHIRVAGKYFADFEIPESMVHLWRYMHHMYRLDAFLQSCPADQDIINHYKLQQSMKMKKHEELETPTFTTSIPIEVNDD

>*Tn*CLIC

MSDEIAENGTANGDVPEIELIIKASTIDGRRKGACLFCQEYFMDLYLLAELKTISLKVTTVDMQKPPPDFRTNFEATHPPILIDNGLAILENEKIERHIMKSVPGGHNLFVQDKEVASLIENLYSKLKLVLVRKDEQKSAALRAHLGRIDGLLERRGTRFLTGDTMCCFDCELMPRLQHIRVAGKYFVDFEIPTSFRALWRYMYHMYQLDAFTQSCPADQDIINHYKLQQALKMKKHEELETPTFTTSIPIDVSENSNAE

>*Pr*CLIC

MSDEIAENGTANGDVPEIELIIKASTIDGRRKGACLFCQEYFMDLYLLAELKTISLKVTTVDMQKPPPDFRTNFEATHPPILIDNGLAILENEKIERHIMKSVPGGHNLFVQDKEVASLIENLYSKLKLVLVRKDEQKSASLRAHLGRIDGLLERRGTRFLTGDTMCCFDCELMPRLQHIRVAGKYFVDFEIPTSFRSLWRYMYHMYQLDAFTQSCPADQDIINHYKLQQALKMKKHEELETPTFTTSIPVDINDSNAEE

>*Dm*CLIC

MSEVESQQSQETNGSSKFDVPEIELIIKASTIDGRRKGACLFCQEYFMDLYLLAELKTISLKVTTVDMQKPPPDFRTNFEATHPPILIDNGLAILENEKIERHIMKNIPGGYNLFVQDKEVATLIENLYVKLKLMLVKKDEAKNNALLSHLRKINDHLSARNTRFLTGDTMCCFDCELMPRLQHIRVAGKYFVDFEIPTHLTALWRYMYHMYQLDAFTQSCPADQDIINHYKLQQSLKMKKHEELETPTFTTYIPIDISE

>*Sfru*CLIC

MSDEIAENGTANGDVPEIELIIKASTIDGRRKGACLFCQEYFMDLYLLAELKTISLKVTTVDMQKPPPDFRTNFEATHPPILIDNGLAILENEKIERHIMKSVPGGHNLFVQDKEVASLIENLYSKLKLVLVRKDEQKSAALRAHLGRIDGLLERRGTRFLTGDTMCCFDCELMPRLQHIRVAGKYFVDFEIPTSFRALWRYMYHMYQLDAFTQSCPADQDIINHYKLQQALKMKKHEELETPTFTTSIPIDVSENSNAE

>*Ar*CLIC

MADDGHENGTANGEVPEIELIIKASTIDGRRKGACLFCQEYFMDLYLLAELKTISLKVTTVDMQKPPPDFRTNFQATPPPILIDNGDAILENEKIERHIMKNVPGGHNLFVQDKEVATLVENLFSKLKLMLLNAKDKDKDPKYSSLMAHLRKIDEHLGRKGTRFLTGDTMCCFDCELMPRLQHIRVAGKYFADFEIPETLVHLWRYMHHMYRLDAFLQSCPADQDIINHYKLQQSMKMKKHEELETPTFTTSIPIEVNDD

>*Mdem*CLIC

MADEGNENGTSNGEVPEIELIIKASTIDGRRKGACLFCQEYFMDLYLLAELKTISLKVTTVDMQKPPPDFRTNFQATPPPILIDGGDAILENEKIERHIMKNIPGGHNLFIQDKEVATLVENLFSKLKLLLLNAKEKDKDPKSSSLMAHLRKIDEHLGRKGTRFLTGDTMCCFDCELMPRLQHIRVAGKYFADFEIPETLVNLWRYMHHMYRLDAFLQSCPADQDIINHYKLQQSMKMKKHEELETPTFTTSIPIEVNDD

>*Oa*CLIC

MADESHDNGTTNGDVPEIELIIKASTIDGRRKGACLFCQEYFMDLYLLAELKTISLKVTTVDMQKPPPDFRTNFQATPPPILIDNGDAILENEKIERHIMKNIPGGHNLFVQDKEVATLVENLYSKLKLLLLNTKDKDKDPKSSSLMAHLRKIDEHLGRKGTRFLTGDTMCCFDCELMPRLQHIRVAGKYFADFEIPGTLVHLWRYMHHMYRLDAFLQSCPADQDIINHYKLQQSMKMKKHEELETPTFTTSIPIEVNDD

>*Cchi*CLIC

SWIKRINAMADEGHENGTSNGEVPEIELIIKASTIDGRRKGACLFCQEYFMDLYLLAELKTISLKVTTVDMQKPPPDFRTNFQATPPPILIDGGDAILENEKIERHIMKNIPGGHNLFIQDKEVATLVENLFSKLKLLLLNAKEKDKDPKSSSLMAHLRKIDEHLGRKGTRFLTGDTMCCFDCELMPRLQHIRVAGKYFADFEIPETLVHLWRYMHHMYRLDAFLQSCPADQDIINHYKLQQSMKMKKHEELETPTFTTSIPIEVNDD

>*Ae*CLIC

MADDNHENGTSNGDVPEIELIIKASTIDGRRKGACLFCQEYFMDLYLLAELKTISLKVTTVDMQKPPPDFRTNFQATPPPILIDNGDAILENEKIERHIMKNIPGGHNLFVQDKEVATLVENLFSKLKLLLLNAKDKDKDPKSSSLMAHLRKIDEHLGRKGTRFLTGDTMCCFDCELMPRLQHIRVAGKYFADFEIPETLVHLWRYMHHMYRLDAFLQSCPADQDIINHYKLQQSMKMKKHEELETPTFTTSIPIEVNDD

>*Aaeg*CLIC

MSDENQENGTRNGDVPEIELIIKASTIDGRRKGACLFCQEYFMDLYLLAELKTISLKVTTVDMQKPPPDFRTNFEATHPPILIDNGLAILENDKIERHIMKSVPGGYNLFVQDKEVATLIENLYSKLKLMLVKKDENKNNALLAHLRKINDHLAARGTRFLTGDTMCCFDCELMPRLQHIRVAGKYFVDFEIPKHLTALWRYMYHMYQLDAFTQSCPADQDIINHYKLQQMLKMKKHEELETPTFTTSIPIDLNDH

>*Tr*CLIC

MADDGHENGTSNGDVPEIELIIKASTIDGRRKGACLFCQEYFMDLYLLAELKTISLKVTTVDMQKPPPDFRTNFQATPPPILIDNGDAILENEKIERHIMKNIPGGHNLFVQDKEVADVVENLFSKLKLLLLNVKEKDKDPKASSLLAHLRKIDKHLGNKGTRFLTGDTMCCFDCELMPRLQHIRVAGKYFANFEIPETFVHLWRYMHHMYRLDAFLQSCPADQDIINHYKLQQSMKMKKHEELETPTFTTSIPIEVNDD

>*Af*CLIC

MTAASTIDGRRKGACLFCQEYFMDLYLLAELKTISLKVTTVDMQKPPPDFRTNFQATPPPILIDNGDAILENEKIERHIMKNIPGGHNLFIQDKDVATLVENLFSKLKLLLLNAKDKDKDPKSSSLMAHLRKIDEHLGRKGTRFLTGDTMCCFDCELMPRLQHIRVAGKYFADFEIPESMVHLWRYMHHMYRLDAFLQSCPADQDIINHYKLQQSMKMKKHEELETPTFTTSIPIEVNDD

>*Aj*CLIC

MDYAKSKEIHRKGEGASTIDGRRKGACLFCQEYFMDLYLLAELKTISLKVTTVDMQKPPPDFRTNFQATPPPILIDNGDAILENEKIERHIMKNIPGGHNLFIQDKDVATLVENLFSKLKLLLLNAKDKDKDPKSSSLMAHLRKIDEHLGRKGTRFLTGDTMCCFDCELMPRLQHIRVAGKYFADFEIPESMVHLWRYMHHMYRLDAFLQSCPADQDIINHYKLQQSMKMKKHEELETPTFTTSIPIEVNDD

>*Acer*CLIC

MADDSHENGTSNGDVPEIELIIKASTIDGRRKGACLFCQEYFMDLYLLAELKTISLKVTTVDMQKPPPDFRTNFQATPPPILIDNGDAILENEKIERHIMKNIPGGHNLFVQDKEVATLVENLFSKLKLLLLNAKDKDKDPKSSSLMAHLRKIDEHLGRKGTRFLTGDTMCCFDCELMPRLQHIRVAGKYFADFEIPETLVHLWRYMHHMYRLDAFLQSCPADQDIINHYKLQQSMKMKKHEELETPTFTTSIPIEVNDD

>*Acep*CLIC

MADDNHENGTSNGDVPEIELIIKASTIDGRRKGACLFCQEYFMDLYLLAELKTISLKVTTVDMQKPPPDFRTNFQATPPPILIDNGDAILENEKIERHIMKNIPGGHNLFVQDKEVATLVENLFSKLKLLLLNAKDKDKDPKSSSLMAHLRKIDEHLGRKGTRFLTGDTMCCFDCELMPRLQHIRVAGKYFADFEIPETLVHLWRYMHHMYRLDAFLQSCPADQDIINHYKLQQSMKMKKHEELETPTFTTSIPIEVNDD

>*Bc*CLIC

MSEVENNQETNGTSNGDVPEIELIIKASTIDGRRKGACLFCQEYFMDLYLLAELKTISLKVTTVDMQKPPPDFRTNFEATHPPILIDNGLAILENDKIERHIMKNIPGGYNLFVQDKEVATLIENLYVKLKLMLVKKDEAKNNALLSHLRKINDHLATRNTRFLTGDTMCCFDCELMPRLQHIRVAGKYFVDFEIPTHFTALWRYMYHMYQLDAFTQSCPADQDIINHYKLQQMLKMKKHEELETPTFTTSIPIDITE

>*Bd*CLIC

MSEVENNQETNGTNNGDVPEIELIIKASTIDGRRKGACLFCQEYFMDLYLLAELKTISLKVTTVDMQKPPPDFRTNFEATHPPILIDNGLAILENDKIERHIMKNIPGGYNLFVQDKEVATLIENLYVKLKLMLVKKDEAKNNALLSHLRKINDHLANRNTRFLTGDTMCCFDCELMPRLQHIRVAGKYFVDFEIPTLFTALWRYMYHMYQLDAFTQSCPADQDIINHYKLQQMLKMKKHEELETPTFTTSIPIDITE

>*Btre*CLIC

MADDGHENGTTNGDVPEIELIIKASTIDGRRKGACLFCQEYFMDLYLLAELKTISLKVTTVDMQKPPPDFRTNFQATPPPILIDNGDAILENEKIERHIMKNIPGGHNLFVQDKEVATLVENIFSKLKLLLINSKDKEMDPKSSSLMTHLRKIDEHLGRKGTRFLTGDTMCCFDCELMPRLQHIRVAGKYFVDFEIPETLVNLWRYMHHMYRLDAFLQSCPADQDIINHYKLQQSMKLKKHEELETPTFTTSIPIEVSDD

>*Bi*CLIC

MADDSHENGTSNGDVPEIELIIKASTIDGRRKGACLFCQEYFMDLYLLAELKTISLKVTTVDMQKPPPDFRTNFQATPPPILIDNGDAILENEKIERHIMKNIPGGHNLFVQDKEVATLVENLFSKLKLLLLNAKDKDKDPKSSSLMAHLRKIDEHLGRKGTRFLTGDTMCCFDCELMPRLQHIRVAGKYFADFEIPETLVHLWRYMHHMYRLDAFLQSCPADQDIINHYKLQQSMKMKKHEELETPTFTTSIPIEVNDD

>*Bter*CLIC

MADDSHENGTSNGDVPEIELIIKASTIDGRRKGACLFCQEYFMDLYLLAELKTISLKVTTVDMQKPPPDFRTNFQATPPPILIDNGDAILENEKIERHIMKNIPGGHNLFVQDKEVATLVENLFSKLKLLLLNAKDKDKDPKSSSLMAHLRKIDEHLGRKGTRFLTGDTMCCFDCELMPRLQHIRVAGKYFADFEIPETLVHLWRYMHHMYRLDAFLQSCPADQDIINHYKLQQSMKMKKHEELETPTFTTSIPIEVNDD

>*Ccin*CLIC

MANDGHENGTSNGDVPEIELIIKASTIDGRRKGACLFCQEYFMDLYLLAELKTISLKVTTVDMQKPPPDFRTNFQATPPPILIDNGDAILENEKIERHIMKNIPGGHNLFVQDKEVATLVENLFSKLKLLLLNAKDKDKDPKSSSLMAHLRKIDEHLGRKGTRFLTGDTMCCFDCELMPRLQHIRVAGKYFADFEIPETLVHLWRYMHHMYRLDAFLQSCPADQDIINHYKLQQSMKMKKHEELETPTFTTSIPIEVNED

>*Csol*CLIC

MADDGQENDTSNGDVPEIELIIKASTIDGRRKGACLFCQEYFMDLYLLAELKTISLKVTTVDMQKPPPDFRTNFQATPPPILIDNGDAILENEKIERHIMKNIPGGYNLFVQDKDVATLVENLFSKLKLLLLNAKDKDKDPKSSSLMAHLRKIDEHLGRKGTRFLTGDTMCCFDCELMPRLQHIRVAGKYFADFEIPESMVHLWRYMHHMYRLDAFLQSCPADQDIINHYKLQQSMKMKKHEELETPTFTTSIPIEVNDD

>*Ci*CLIC

MADDGHENGSSNGQVPQIELIIKASTIDGRRKGACLFCQEYFMDLYLLAELKTISLKVTTVDMQKPPPDFRTNFQATPPPILIDNGDAILENEKIERHIMKNIPGGHNLFVQDKEVATLVENLFSKLKLLLLNAKEKDKDPKSSSLMAHLRKIDEHLGRKGTRFLTGDTMCCFDCELMPRLQHIRVAGKYFADFEIPESLVHLWRYMHHMYRLDAFLQSCPADQDIINHYKLQQSMKMKKHEELETPTFTTSIPIEVNDDE

>*Cf*CLIC

MADDSHENGTSNGDVPEIELIIKASTIDGRRKGACLFCQEYFMDLYLLAELKTISLKVTTVDMQKPPPDFRTNFQATPPPILIDNGDAILENEKIERHIMKNIPGGHNLFIQDKDVATLVENVFSKLKLLLLNAKDKDKDPKSSSLMAHLRKIDDHLGRKGTRFLTGDTMCCFDCELMPRLQHIRVAGKYFADFEIPESMVHLWRYMHHMYRLDAFLQSCPADQDIINHYKLQQSMKMKKHEELETPTFTTSIPIEVNDE

>*Cv*CLIC

MDLYLLAELKTISLKVTTVDMQKPPPDFRTNFQATPPPILIDGGDAILENEKIERHIMKNIPGGHNLFIQDKEVATLVENLFSKLKLLLLNAKEKDKDPKSSSLMAHLRKIDEHLGRKGTRFLTGDTMCCFDCELMPRLQHIRVAGKYFADFEIPETLVHLWRYMHHMYRLDAFLQSCPADQDIINHYKLQQSMKMKKHEELETPTFTTSIPIEVNDD

>*Cp*CLIC

MSDENQENGTRNGQEVPEIELIIKASTIDGRRKGACLFCQEYFMDLYLLAELKTISLKVTTVDMQKPPPDFRTNFEATHPPILIDNGLAILENDKIERHIMKSVPGGYNLFVQDKEVATLIENLYSKLKLMLVKKDENKNNALLAHLRKINDHLAARGTRFLTGDTMCCFDCELMPRLQHIRVAGKYFVDFEIPKHLTALWRYMYHMYQLDAFTQSCPADQDIINHYKLQQMLKMKKHEELETPTFTTSIPIDLNDH

>*Da*CLIC

MADEAHENGTSNGDVPEIELIIKASTIDGRRKGACLFCQEYFMDLYLLAELKTISLKVTTVDMQKPPPDFRTNFQATPPPILIDNGDAILENEKIERHIMKNIPGGHNLFVQDKEVATLVENLFTKLKLLLVNAKDKDKDPKSSSLMAHLRKIDEHLGRKGTRFLTGDTMCCFDCELMPRLQHIRVAGKYFADFEIPETLVNLWRYMHHMYRLDAFLQSCPADQDIINHYKLQQSMKMKKHEELETPTFTTSIPIEVNDD

>*Dn*CLIC

MADDGHENGTSNGDVPEIELIIKASTIDGRRKGACLFCQEYFMDLYLLAELKTISLKVTTVDMQKPPPDFRTNFQATPPPILIDNGDAILENEKIERHIMKNIPGGHNLFVQDKEVATLVENLFSKLKLLLLNAKDKDKDPKSSSLMAHLRKIDEHLGRKGTRFLTGDTMCCFDCELMPRLQHIRVAGKYFADFEIPETLVHLWRYMHHMYRLDAFLQSCPADQDIINHYKLQQSMKMKKHEELETPTFTTSIPIEVNDD

>*Em*CLIC

MADDSHENGTSNGDVPEIELIIKASTIDGRRKGACLFCQEYFMDLYLLAELKTISLKVTTVDMQKPPPDFRTNFQATPPPILIDNGDAILENEKIERHIMKNIPGGHNLFVQDKEVATLVENLFSKLKLLLLNAKDKDKDPKSSSLMAHLRKIDEHLGRKGTRFLTGDTMCCFDCELMPRLQHIRVAGKYFADFEIPETLVHLWRYMHHMYRLDAFLQSCPADQDIINHYKLQQSMKMKKHEELETPTFTTSIPIEVNDD

>*Fa*CLIC

MADEAHDNGTSNGDVPEIELIIKASTIDGRRKGACLFCQEYFMDLYLLAELKTISLKVTTVDMQKPPPDFRTNFQATPPPILIDNGDAILENEKIERHIMKNIPGGHNLFVQDKEVATLVENLFTKLKLLFLNAKEKDKDPKSSSLMAHLRKIDEHLGRKGTRFLTGDTMCCFDCELMPRLQHIRVAGKYFADFEIPETLVNLWRYMHHMYRLDAFLQSCPADQDIINHYKLQQSMKMKKHEELETPTFTTSIPIEVNDD

>*Fe*CLIC

MADDNHENGTGNGDVPEIELIIKASTIDGRRKGACLFCQEYFMDLYLLAELKTISLKVTTVDMQKPPPDFRTNFQATPPPILIDNGDAILENEKIERHIMKNIPGGHNLFIQDKEVATLVENLFSKLKLLLLNAKDKDKDPKSSSLMAHLRKIDEHLGRKGTRFLTGDTMCCFDCELMPRLQHIRVAGKYFADFEIPETLVHLWRYMHHMYRLDAFLQSCPADQDIINHYKLQQSMKMKKHEELETPTFTTSIPIEVNDD

>*Gfla*CLIC

MDLYLLAELKTISLKVTTVDMQKPPPDFRTNFQATPPPILIDNGDAILENEKIERHIMKNIPGGHNLFVQDKEVATLVENLFSKLKLLLLNAKDKDKDPKSSSLMAHLRKIDEHLGRKGTRFLTGDTMCCFDCELMPRLQHIRVAGKYFADFEIPVTLVHLWRYMHHMYRLDAFLQSCPADQDIINHYKLQQSMKMKKHEELELPTFTTSIPIEVNDD

>*Hl*CLIC

MADDSHENGTSNGDVPEIELIIKASTIDGRRKGACLFCQEYFMDLYLLAELKTISLKVTTVDMQKPPPDFRTNFQATPPPILIDNGDAILENEKIERHIMKNIPGGHNLFVQDKEVATLVENLFSKLKLLLLNAKDKDKDPKSSSLMAHLRKIDEHLGRKGTRFLTGDTMCCFDCELMPRLQHIRVAGKYFADFEIPETLVHLWRYMHHMYRLDAFLQSCPADQDIINHYKLQQSMKMKKHEELETPTFTTSIPIEVNDD

>*Hm*CLIC

MSDEIAENGTANGDVPEIELIIKASTIDGRRKGACLFCQEYFMDLYLLAELKTISLKVTTVDMQKPPPDFRTNFEATHPPILIDNGLAILENEKIERHIMKSVPGGHNLFVQDKEVASLIENLYSKLKLVLVRKDEQKSAALRSHLQRIDALLERRGTRFLTGDTMCCFDCELMPRLQHIRVAGKYFVDFEIPASFRALWRYMHHMYQLDAFAQSCPADQDIINHYKLQQLATKGLIMSPTLTRQRKEEPTALKMKKHEELETPTFTTSIPVDVDDISNNKQ

>*Harm*CLIC

MSDEIAENGTANGDVPEIELIIKASTIDGRRKGACLFCQEYFMDLYLLAELKTISLKVTTVDMQKPPPDFRTNFEATHPPILIDNGLAILENEKIERHIMKSVPGGHNLFVQDKEVASLIENLYSKLKLVLVRKDEQKSAALRAHLGRIDGLLERRGTRFLTGDTMCCFDCELMPRLQHIRVAGKYFVDFEIPTSFRALWRYMYHMYQLDAFTQSCPADQDIINHYKLQQALKMKKHEELETPTFTTSIPIDVSENSNAE

>*Hh*CLIC

MMADEAHENGTSNGDVPEIELIIKASTIDGRRKGACLFCQEYFMDLYLLAELKTISLKVTTVDMQKPPPDFRTNFQATPPPILIDNGDAILENEKIERHIMKNIPGGHNLFVQDKEVATLVENLFSKLKLLLLNAKDKDKDPKSSSLMAHLRKIDEHLGRKGTRFLTGDTMCCFDCELMPRLQHIRVAGKYFADFEIPETLVHLWRYMHHMYRLDAFLQSCPADQDIINHYKLQQSMKMKKHEELETPTFTTSIPIEVNDD

>*Ld*CLIC

MADEVSENGHAENGDVPEIELIIRASTIDGRRKGACLFCQEYFMELYLLAELKTISLKVTTVDMQKPPPDFRTNFEATPPPILIDRGMAILENEKIERHIMKNVPGGHNLFVQDKEVATLIENLYTKFKMYVRKYESPDTSESRQPLLSHLERINDFLAKRGTRFLTGDTMSCFDCELMPRLQHIRIGAKAFRKFEIPTTFTALWTYMANMYELEAFIQSCPADQDIISHIKNQLGLRTTARIELETPVYSTAIPVLAES

>*Lf*CLIC

MADEVQENGSSNGEVPEIELIIKASTIDGRRKGACLFCQEYFMDLYLLAELKTISLKVTTVDMQKPPPDFRTNFQATPPPILIDNGDAILENEKIERHIMKNIPGGHNLFVQDKEVATLVENLFSKLKLLLLNAKEKDKDPKSSSLMAHLRKINEHLGRKGTRFLTGETMCCFDCELMPRLQHIRVAGKYFANFEIPENLVNLWRYMHHMYRLDAFLQSCPADQDIINHYKLQQSMKMKKHEELETPTFTTSIPIEVNDD

>*Macin*CLIC

MADAKTVQNGTNVPEIELIIKASTIDGRRKGACLFCHEYFMDLYLLAEMKTISLKVTTVDMQKPPADFRSNFEAHQPPILIDGQKALTDNPEIERHIMKVIPGGHNLFIVDKEVATLIENLFSKLKLLLLNAKDKEKDPKKSALMSHLRKIDEYLGRKGTRFLTGDTMCCFDCELMPRLQHIRVAGKYFADFEIPDTMIYLWRYMHTMYQLDAFTQSSPADQDIINHYKLQQSIKMKKHEELETPTFTTSIPANIIPFPENYSAPIVDIPLNDEPDNDSTDKETANKNFDYVNNEKIENAGP

>*Ms*CLIC

MSDEIAENGTANGDVPEIELIIKASTIDGRRKGACLFCQEYFMDLYLLAELKTISLKVTTVDMQKPPPDFRTNFEATHPPILIDNGLAILENEKIERHIMKSVPGGHNLFVQDKEVASLIENLYSKLKLVLVRKDEQKSAALRAHLGRIDALLERRGTRFLTGDTMCCFDCELMPRLQHIRVAGKYFVDFEIPTSFRALWRYMYHMYQLDAFTQSCPADQDIINHYKLQQALKMKKHEELETPTFTTSIPVDVNEQAAE

>*Mr*CLIC

MADDSHENGTSNGDVPEIELIIKASTIDGRRKGACLFCQEYFMDLYLLAELKTISLKVTTVDMQKPPPDFRTNFQATPPPILIDNGDAILENEKIERHIMKNIPGGHNLFVQDKEVATLVENLFSKLKLLLLNAKDKDKDPKSSSLMAHLRKIDEHLGRKGTRFLTGDTMCCFDCELMPRLQHIRVAGKYFADFEIPETLVHLWRYMHHMYRLDAFLQSCPADQDIINHYKLQQSMKMKKHEELETPTFTTSIPIEVNDD

>*Nlec*CLIC

MADDGHENGTANGDVPEIELIIKASTIDGRRKGACLFCQEYFMDLYLLAELKTISLKVTTVDMQKPPPDFRTNFQATPPPILIDNGDAILENEKIERHIMKNVPGGHNLFVQDKEVATLVENLFSKLKLMLLNAKDKDKDPKYSSLMSHLRKIDEHLGRKGTRFLTGDTMCCFDCELMPRLQHIRVAGKYFADFEIPETLVHLWRYMHHMYRLDAFLQSCPADQDIINHYKLQQSMKMKKHEELETPTFTTSIPIEVNDD

>*Nlug*CLIC

MSEDTNQNGTENGSVTEIELIIKASTIDGRRKGACLFCQEYFMDLYLLAELKTISLKVTTVDMQKPPPDFRTNFEATPPPILIDNGLAVLENEKIERHIMKNVPGGHNLFVQDKEVATLIENLYSKLKLMLLKKDDVSTNSLMSHLRKIDDHLGRKNTRFLTGDTMCCFDCELMPRLQHIRVAGKYFRDFEIPSELKYLWRYMLHMYQLQAFTQSCPADQDIINHYKLQQGMKMKKHEELETPTFTTSIPIEPSDLDLTDD

>*Ob*CLIC

MADDSHENGTSNGDVPEIELIIKASTIDGRRKGACLFCQEYFMDLYLLAELKTISLKVTTVDMQKPPPDFRTNFQATPPPILIDNGDAILENEKIERHIMKNIPGGHNLFVQDKEVATLVENLFSKLKLLLLNAKDKDKDPKSSSLMAHLRKIDEHLGRKGTRFLTGDTMCCFDCELMPRLQHIRVAGKYFADFEIPETLVHLWRYMHHMYRLDAFLQSCPADQDIINHYKLQQSMKMKKHEELETPTFTTSIPIEVNDD

>*Pb*CLIC

MADDNHENGTSNGDVPEIELIIKASTIDGRRKGACLFCQEYFMDLYLLAELKTISLKVTTVDMQKPPPDFRTNFQATPPPILIDNGDAILENEKIERHIMKNIPGGHNLFVQDKEVATLVENLFSKLKLLLLNAKDKDKDPKSSSLMAHLRKIDEHLGRKGTRFLTGDTMCCFDCELMPRLQHIRVAGKYFADFEIPETLVHLWRYMHHMYRLDAFLQSCPADQDIINHYKLQQSMKMKKHEELETPTFTTSIPIEVNDD

>*Pc*CLIC

MADDGHENGTSNGDVPEIELIIKASTIDGRRKGACLFCQEYFMDLYLLAELKTISLKVTTVDMQKPPPDFRTNFQATPPPILIDNGDAILENEKIERHIMKNIPGGHNLFVQDKEVATLVENLFSKLKLLLLNAKDKDKDPKSSSLMAHLRKIDEHLGRKGTRFLTGDTMCCFDCELMPRLQHIRVAGKYFANFEIPESLVHLWRYMHHMYRLDAFLQSCPADQDIINHYKLQQSMKMKKHEELETPTFTTSIPIEVNDD

>*Pd*CLIC

MADDGHENGTSNGDVPEIELIIKASTIDGRRKGACLFCQEYFMDLYLLAELKTISLKVTTVDMQKPPPDFRTNFQATPPPILIDNGDAILENEKIERHIMKNIPGGHNLFVQDKEVATLVENLFSKLKLLLLNAKDKDKDPKSSSLMAHLRKIDEHLGRKGTRFLTGDTMCCFDCELMPRLQHIRVAGKYFANFEIPESLVHLWRYMHHMYRLDAFLQSCPADQDIINHYKLQQSMKMKKHEELETPTFTTSIPIEVNDD

>*Si*CLIC

MADDNHENGTSNGDVPEIELIIKASTIDGRRKGACLFCQEYFMDLYLLAELKTISLKVTTVDMQKPPPDFRTNFQATPPPILIDNGDAILENEKIERHIMKNIPGGHNLFVQDKEVATLVENLFSKLKLLLLNAKDKDKDPKSSSLMAHLRKIDEHLGRKGTRFLTGDTMCCFDCELMPRLQHIRVAGKYFADFEIPETLVHLWRYMHHMYRLDAFLQSCPADQDIINHYKLQQSMKMKKHEELETPTFTTSIPIEVNDD

>*Tp*CLIC

MADDGHENGTSNGDVPEIELIIKASTIDGRRKGACLFCQEYFMDLYLLAELKTISLKVTTVDMQKPPPDFRTNFQATPPPILIDNGDAILENEKIERHIMKNIPGGHNLFVQDKDVATLVENLFSKLKLLLLNAKDKDKDVKSSSLMAHLRKIDEHLGRKGTRFLTGDTMCCFDCELMPRLQHIRVAGKYFADFDIPESMVNLWRYMHHMYRLDAFLQSCPADQDIINHYKLQQSMKMKKHEELETPTFTTSIPIEVNED

>*Vcan*CLIC

MADEGHENGTSNGDVPEIELIIKASTIDGRRKGACLFCQEYFMDLYLLAELKTISLKVTTVDMQKPPPDFRTNFQATPPPILIDNGESILENEKIERHIMKNIPGGHNLFVQDKEVATLVENLFSKLKLLLLNAKEKDKDPKSSSLMAHLRKIDEHLGRKGTRFLTGDTMCCFDCELMPRLQHIRVAGKYFADFEIPETLVNLWRYMHHMYRLDAFLQSCPADQDIINHYKLQQSMKMKKHEELETPTFTTSIPIEVNDD

>*Vm*CLIC

MADTDGHENGTSNGDVPEIELIIKASTIDGRRKGACLFCQEYFMDLYLLAELKTISLKVTTVDMQKPPPDFRTNFQATPPPILIDNGDAILENEKIERHIMKNIPGGHNLFVQDKEVATLVENLFSKLKLLLLNAKDKDKDPKSSSLMAHLRKIDEHLGRKGTRFLTGDTMCCFDCELMPRLQHIRVAGKYFANFEIPESLVHLWRYMHHMYRLDAFLQSCPADQDIINHYKLQQSMKMKKHEELETPTFTTSIPIEVNDD

>*Sl*CLIC

MDLYLLAELKTISLKVTTVDMQKPPPDFRTNFEATHPPILIDNGLAILENEKIERHIMKSVPGGHNLFVQDKEVASLIENLYSKLKLVLVRKDEQKSAALRAHLGRIDGLLERRGTRFLTGDTMCCFDCELMPRLQHIRVAGKYFVDFEIPTSFRALWRYMYHMYQLDAFTQSCPADQDIINHYKLQQALKMKKHEELETPTFTTSIPIDVSENSNAE

>*Pxyl*CLIC

MSEEIAENGTTNGDVPEIELIIKASTIDGRRKGACLFCQEYFMDLYLLAELKTISLKVTTVDMQKPPPDFRTNFEATHPPILIDNGLAILENEKIERHIMKSVPGGHNLFVQDKEVASLIENLYSKLKLVLVRKDEHKSASLLAHLGRIDALLARRATRFLTGDTMCCFDCELMPRLQHIRVAGKYFVDFEIPTSLSALWRYMHHMYQLDAFTQSCPADQDIINHYKLQQALKMKKHEELETPTFTTSIPISITDNNGDQ

>*Pxut*CLIC

MSDEIVENGTANGDVPEIELIIKASTIDGRRKGACLFCQEYFMDLYLLAELKTISLKVTTVDMQKPPPDFRTNFEATHPPILIDNGLAILENEKIERHIMKSVPGGHNLFVQDKEVASLIENLYSKLKLVLVRKDEQKSAALRAHLGRIDALLERRATRFLTGDTMCCFDCELMPRLQHIRVAGKYFVDFEIPTSFRALWRYMYHMYQLDAFTQSCPADQDIINHYKLQQALKMKKHEELETPTFTTSIPIDVNDLSATE

>*Pa*CLIC

MSDEIAENGTANGDVPEIELIIKASTIDGRRKGACLFCQEYFMDLYLLAELKTISLKVTTVDMQKPPPDFRTNFEATHPPILIDNGLAILENEKIERHIMKSVPGGHNLFVQDKEVASLIENLYSKLKLVLVRKDEQKSAALRAHLGRIDALLERRGTRFLTGDTMCCFDCELMPRLQHIRVAGKYFVDFEIPTSFRALWRYMYHMYQLDAFTQSCPADQDIINHYKLQQALKMKKHEELETPTFTTSIPIDVNDLNNSEQ

>*Ppol*CLIC

MSDEIAENGTANGDVPEIELIIKASTIDGRRKGACLFCQEYFMDLYLLAELKTISLKVTTVDMQKPPPDFRTNFEATHPPILIDNGLAILENEKIERHIMKSVPGGHNLFVQDKEVASLIENLYSKLKLVLVRKDEQKSAALRAHLGRIDALLERRATRFLTGDTMCCFDCELMPRLQHIRVAGKYFVDFEIPTSFRALWRYMYHMYQLDAFTQSCPADQDIINHYKLQQALKMKKHEELETPTFTTSIPIDVNDVSGAE

>*Pm*CLIC

MSDEIAENGTANGDVPEIELIIKASTIDGRRKGACLFCQEYFMDLYLLAELKTISLKVTTVDMQKPPPDFRTNFEATHPPILIDNGLAILENEKIERHIMKSVPGGHNLFVQDKEVASLIENLYSKLKLVLVRKDEQKSAALRAHLGRIDALLERRATRFLTGDTMCCFDCELMPRLQHIRVAGKYFVDFEIPTNFRALWRYMYHMYQLDAFTQSCPADQDIINHYKLQQALKMKKHEELETPTFTTSIPIDVNDLSATE

>*Mecin*CLIC

MDLYLLAELKTISLKVTTVDMQKPPPDFRTNFEATHPPILIDNGLAILENEKIERHIMKSVPGGHNLFVQDKEVASLIENLYSKLKLVLVRKDEQKSAALRAHLQRIDALLERRGTRFLTGDTMCCFDCELMPRLQHIRVAGKYFVDFEIPTSFRALWRYMYHMYQLDAFTQSCPADQDIINHYKLQQALKMKKHEELETPTFTTSIPIDVNDVNNSE

>*Hk*CLIC

MSDEIAENGTVNGDVPEIELIIKASTIDGRRKGACLFCQEYFMDLYLLAELKTISLKVTTVDMQKPPPDFRTNFEATHPPILIDNGLAILENEKIERHIMKSVPGGHNLFVQDKEVASLIENLYSKLKLVLVRKDEQKSAALRAHLGRIDALLERRNTRFLTGDTMCCFDCELMPRLQHIRVAGKYFVDFEIPTTFRALWRYMYHMYQLDAFTQSCPADQDIINHYKLQQALKMKKHEELETPTFTTSIPIDVSELNE

>*Hz*CLIC

MSDEIAENGTANGDVPEIELIIKASTIDGRRKGACLFCQEYFMDLYLLAELKTISLKVTTVDMQKPPPDFRTNFEATHPPILIDNGLAILENEKIERHIMKSVPGGHNLFVQDKEVASLIENLYSKLKLVLVRKDEQKSAALRAHLGRIDGLLERRGTRFLTGDTMCCFDCELMPRLQHIRVAGKYFVDFEIPTSFRALWRYMYHMYQLDAFTQSCPADQDIINHYKLQQALKMKKHEELETPTFTTSIPIDVSENSNAE

>*Gm*CLIC

MSDEIAENGTANGDVPEIELIIKASTIDGRRKGACLFCQEYFMDLYLLAELKTISLKVTTVDMQKPPPDFRTNFEATHPPILIDNGLAILENEKIERHIMKSVPGGHNLFVQDKEVASLIENLYSKLKLVLVRKDEQKSAALRAHLGRIDALLERRGTRFLTGDTMCCFDCELMPRLQHIRVAGKYFVDFEIPTSFRALWRYMYHMYQLDAFTQSCPADQDIINHYKLQQALKMKKHEELETPTFTTSIPIDVNDLNNTE

>*Dple*CLIC

MMSDEIAENGTANGDVPEIELIIKASTIDGRRKGACLFCQEYFMDLYLLAELKTISLKVTTVDMQKPPPDFRTNFEATHPPILIDNGLAILENEKIERHIMKSVPGGHNLFVQDKEVASLIENLYSKLKLVLVRKDEHKSAALRAHLARIDALLERRGTRFLTGDTMCCFDCELMPRLQHIRVAGKYFVDFEIPTSFRALWRYMYHMYQLDAFTQSCPADQDIINHYKLQQALKMKKHEELETPTFTTSIPVDVNEPNEQ

>*Bman*CLIC

MSDEIAENGTANGDVPEIELIIKASTIDGRRKGACLFCQEYFMDLYLLAELKTISLKVTTVDMQKPPPDFRTNFEATHPPILIDNGLAILENEKIERHIMKSVPGGHNLFVQDKEVASLIENLYSKLKLVLVRKDEQKSAALRAHLGRIDGLLERRETRFLTGDTMCCFDCELMPRLQHIRVAGKYFVDFEIPTTFRALWRYMYHMYQLDAFTQSCPADQDIINHYKLQQALKMKKHEELETPTFTTSIPIDVNELNNAE

>*Bmor*CLIC

MSDEIAENGTANGDVPEIELIIKASTIDGRRKGACLFCQEYFMDLYLLAELKTISLKVTTVDMQKPPPDFRTNFEATHPPILIDNGLAILENEKIERHIMKSVPGGHNLFVQDKEVASLIENLYSKLKLVLVRKDEQKSAALRAHLGRIDGLLERRETRFLTGDTMCCFDCELMPRLQHIRVAGKYFVDFEIPTTFRALWRYMYHMYQLDAFTQSCPADQDIINHYKLQQALKMKKHEELETPTFTTSIPIDVNELNNAE

>*Ba*CLIC

MSDEIAENGTANGDVPEIELIIKASTIDGRRKGACLFCQEYFMDLYLLAELKTISLKVTTVDMQKPPPDFRTNFEATHPPILIDNGLAILENEKIERHIMKSVPGGHNLFVQDKEVASLIENLYSKLKLVLVRKDEQKSAALRAHLGRIDALLERRGTRFLTGDTMCCFDCELMPRLQHIRVAGKYFVDFEIPTSFRALWRYMFHMYQLDAFTQSCPADQDIINHYKLQQALKMKKHEELETPTFTTSIPIDFNDLNNSDQ

>*Atra*CLIC

MSDEIAENGTTNGDVPEIELIIKASTIDGRRKGACLFCQEYFMDLYLLAELKTISLKVTTVDMQKPPPDFRTNFEATHPPILIDNGLAILENEKIERHIMKSVPGGHNLFVQDKEVASLIENLYSKLKLVLVRKDEQKSAALRAHLGRIDALLERRETRFLTGDTMCCFDCELMPRLQHIRVAGKYFVDFEIPTSFRALWRYMYHMYQLDAFTQSCPADQDIINHYKLQQALKMKKHEELETPTFTTSIPIDVNDLNNTE

>*Tc*CLIC

MSDEVAENGHSEGDVPEIELIIRASTIDGRRKGACLFCQEYFMELYLLAELKTISLKVTTVDMQKPPPDFRTNFEATPPPILIDRGMAILENEKIERHIMKSVPGGHNLFVQDKEVATLIENLYTKFKMYVTKFESTDTKEASQPLLSHLERINDFLAKRGTRFLTGDTMSCFDCELMPRLQHIRIGAKAFRKFEIPTRFTALWTYMANMYELEAFRQSCPADQDIISHIKNQLGLRTTARIELETPVFTTSIPIVAET

>*Atum*CLIC

MADEVSQNGTSGLSDEEVPEIELIIRASTIDGRRKGACLFCQEYFMELYLLAELKTISLKVTTVDMQKPPPDFRTNFEATPPPILIDRGMAILENEKIERHIMKTVPGGHNLFVQDKEVATLIENIYTKFKSYVTKFVSDGKEDSTPNPPLLHHLERINDFLAKRGTRFLTGDTMSCFDCELMPRLQHIRIGAKAFRKFEIPTRLTALWRYMSNMYELEAFRQSCPADQDIISHIKNQLGIKTTARIELETPVFTTSIPILSES

>*Apla*CLIC

MSDEITENGHENGDVPEIELIIKASTIDGRRKGACLFCQEYFMDLYLLAELKTISLKVTTVDMQKPPPDFRTNFEATPPPILIDRGMAILENEKIERHIMKNIPGGHNLFVQDKEVATLIENLYSKLKLVLVKKDEQKSAQLLAHLKRIDEHLRRRDTRFLTGDTICCFDCELMPRLQHIRVAAKYFVDFDIPKELTSLWRYMYNMYQLDAFTQSCPADQDIINHYKIQQGLKMKKHEELETPTFTTSIPIDVSQLN

>*Dpon*CLIC

MADEVNENGHPENGDVPEIELIIRASTIDGRRKGACLFCQEYFMELYLLAELKTISLKVTTVDMQKPPPDFRTNFEATPPPILIDRGMAILENEKIERHIMKTVPGGHNLFVQDKEVATLIENLYTKFKMYVTKYESADTKEASQPLLSHLERINDFLVKRATRFLTGDTMSCFDCELMPRLQHIRIGAKAFRKFEIPTKFTGLWRYMENMYELEAFRQSCPADQDIISHIKNQLGLRTTARIELETPIYTTSIPILDS

>*Dv*CLIC

MADEVAENGHAENGDVPEIELIIRASTIDGRRKGACLFCQEYFMELYLLAELKTISLKVTTVDMQKPPPDFRTNFEATPPPILIDRGMAILENEKIERHIMKNVPGGHNLFVQDKEVATLIENLYTKFKTYVRKFESTDTTENRQPLYSHLERINDFMAKRGTRFLTGDTMSCFDCELMPRLQHIRIGAKAFRKFEIPSTFKALWNYMANMYELEAFIQSCPADQDIISHIKNQLGLKTTARIELETPVYTTAIPVLSES

>*Haxy*CLIC

MSDDIVENGHEENGDVPEIELIIRASTIDGRRKGACLFCHEYFMELYLLAELKTISLKVTTVDMQKPPPDFRTNFEATPPPILIDRGLAILENEKIERHIMKSVPGGHNLFVQDKEVATLIENLYTKFKIYITKYESTDTNEASQPLLSQLMKINDFLAKRATRFLTGDTMSCFDCELMPRLQHIRIGAKAFRNFEIPTKFSALWRYMFNMYELEAFRQSCPADQDIISHIKNQLGLRTKARIELETPVYSTSIPILNEA

>*Nves*CLIC

MSEEIAENGHANGGDVPEIELIIKASTIDGRRKGACLFCQEYFMDLYLLAELKTISLKVTTVDMQKPPPDFRTNFEATPPPILIDRGMAILENEKIERHIMKNVPGGHNLFVQDKEVATLIENVYGKLKLVLVKKDDKKNAAPLLAHLKRIDEHLRKRDTRFLTGDTMCCFDCELMPRLQHIRVAAKYFLDFDIPKELTSLWRYMYHMYQLDAFTQSCPADQDIINHYKLQQALKMNKHEELETPTFTTSIPIDVSQ

>*Ot*CLIC

MSDEIPENGHENGEDVPVVELIIKASTIDGVRKGACLFCQEYFMDLYLLAELKTISLKVTTVDMQKPPPDFRTNFEATPPPILIDRGTAILENEKIERYIMKNVPGGYNLFVQDKEVATLIENLYSKLKLILVKKDEKKNNQLTSHLKRINEHLQKRETRFLTGDAICCFDCELMPRLQHIRVAGKYFVDYEIPEDFVALWRYMYHMYQLDAFTQSCPADQDIINHYKLQQAMKMKKHEELELPTYTTSIPVDIGDVQ

>*So*CLIC

MADEIQENGHAENGDVPEIELIIRASTIDGRRKGACLFCQEYFMELYLLAELKTISLKVTTVDMQKPPPDFRTNFEATPPPILIDRGMAILENEKIERHIMKTVPGGHNLFVQDKEVATLIENLYTKFKMYVTKYESADTKEASQPLFSHLERINDFLVKRGTRFLTGDTMSCFDCELMPRLQHIRIGAKAFRKFEIPTRFQGLWRYMENMYELEAFRQSCPADQDIISHIKNQLGLRTTARIELETPVYTTSIPILDS

>*Bo*CLIC

MSEVENNQETNGTNNGDVPEIELIIKASTIDGRRKGACLFCQEYFMDLYLLAELKTISLKVTTVDMQKPPPDFRTNFEATHPPILIDSGLAILENDKIERHIMKNIPGGYNLFVQDKEVATLIENLYVKLKLMLVKKDEAKNNALLSHLRKINDHLANRNTRFLTGDTMCCFDCELMPRLQHIRVAGKYFVDFEIPTHFTALWRYMYHMYQLDAFTQSCPADQDIINHYKLQQMLKMKKHEELETPTFTTSIPIDITE

>*Ccap*CLIC

MYCNCLKPVSSVFCELTKGKCLVAATFVLRLVSANFIHTSRVFKMSEVENNQETNGTSNGDVPEIELIIKASTIDGRRKGACLFCQEYFMDLYLLAELKTISLKVTTVDMQKPPPDFRTNFEATHPPILIDNGLAILENDKIERHIMKNIPGGYNLFVQDKEVATLIENLYVKLKLMLVKKDEAKNNALLSHLRKINDHLANRNTRFLTGDTMCCFDCELMPRLQHIRVAGKYFVDFEIPTHFTALWRYMYHMYQLDAFTQSCPADQDIINHYKLQQMLKMKKHEELETPTFTTSIPIDITE

>*Cq*CLIC

MSDENQENGTRNGQEVPEIELIIKASTIDGRRKGACLFCQEYFMDLYLLAELKTISLKVTTVDMQKPPPDFRTNFEATHPPILIDNGLAILENDKIERHIMKSVPGGYNLFVQDKEVATLIENLYSKLKLMLVKKDENKNNALLAHLRKINDHLAARGTRFLTGDTMCCFDCELMPRLQHIRVAGKYFVDFEIPKHLTALWRYMYHMYQLDAFTQSCPADQDIINHYKLQQMLKMKKHEELETPTFTTSIPIDLNDH

>*Gfus*CLIC

MSEAEHAETNGTANGDVPEIELIIKASTIDGRRKGACLFCQEYFMDLYLLAELKTISLKVTTVDMQKPPPDFRTNFEATHPPILIDNGLAILENDKIERHIMKNIPGGYNLFVQDKEVATLIENLYVKLKLMLVKKDEAKNNALLAHLKKINDHLANRNTRFLTGDTMCCFDCELMPRLQHIRVAGKYFVDFEIPTHLTALWRYMYHMYQLDAFTQSCPADQDIINHYKLQQMLKMKKHEELETPTFTTSIPVDIAD

>*Hi*CLIC

MSEDNQQENGTANGDVPEIELIIKASTIDGRRKGACLFCQEYFMDLYLLAELKTISLKVTTVDMQKPPPDFRTNFEATHPPILIDNGLAILENDKIERHIMKNIPGGYNLFVQDKEVATLIENLYSKLKLMLVKKDESKNNALLSHLRKINDHLASRGTRFLTGDTICCFDCELMPRLQHIRVAGKYFVDFEIPTHLTALWRYMYHMYQLDAFTQSCPADQDIINHYKLQQMLKMKKHEELETPTFTTSIPIDITE

>*Mdom*CLIC

MSEAELQETNGTSEVPEIELIIKASTIDGRRKGACLFCQEYFMDLYLLAELKTISLKVTTVDMQKPPPDFRTNFEATHPPILIDNGLAILENDKIERHIMKSIPGGYNLFVQDKEVATLIENLYVKLKLMLVKKDEAKNNALLSHLKKINDHLANRNTRFLTGDTMCCFDCELMPRLQHIRVAGKYFVDFEIPTHYTALWRYMYHMYQLDAFTQSCPADQDIINHYKLQQMLKMKKHEELETPTFTTSIPIDISE

>*Sc*CLIC

MSEAELQETNGGTSNGDVPEIELIIKASTIDGRRKGACLFCQEYFMDLYLLAELKTISLKVTTVDMQKPPPDFRTNFEATHPPILIDNGLAILENDKIERHIMKNIPGGYNLFVQDKEVATLIENLYVKLKLMLVKKDDAKNNALLSHLKKINDHLANRNTRFLTGDTMCCFDCELMPRLQHIRVAGKYFVDFEIPTHYTALWRYMYHMYQLDAFTQSCPADQDIINHYKLQQMLKMKKHEELETPTFTTSIPIDISE

>*Zc*CLIC

MSEVENNQETNGTSNGDVPEIELIIKASTIDGRRKGACLFCQEYFMDLYLLAELKTISLKVTTVDMQKPPPDFRTNFEATHPPILIDNGLAILENDKIERHIMKNIPGGYNLFVQDKEVATLIENLYVKLKLMLVKKDEAKNNALLSHLRKINDHLATRNTRFLTGDTMCCFDCELMPRLQHIRVAGKYFVDFEIPTHFTALWRYMYHMYQLDAFTQSCPADQDIINHYKLQQMLKMKKHEELETPTFTTSIPIDITE

>*Apis*CLIC

MADEDAPHANGNGHEENGHVPEIELIIKASTIDGRRKGACLFCQEYFMDLYLLAELKTISLKVTTVDMQKPPPDFRSNFDANPPPILIDNGMAVLENEKIERHIMKNVPGGYNLFVQDKEVATLIENLYSKLKLMLLKKDDVSINSLLSHLRKINLHLEKKNTRFLTGDTMCCFDCELMPRLQHIRVAGKYFMDFQMPTDLRYLWRYMLHMYQLDAFTQSCPADQDIVNHYKQQQNVKMKKHEELETPTFTTSIPVDLDLQQDE

>*Ag*CLIC

MADEDAPHANGNVHEENGHVPEIELIIKASTIDGRRKGACLFCQEYFMDLYLLAELKTISLKVTTVDMQKPPPDFRSNFDANPPPILIDNGMAVLENEKIERHIMKNVPGGYNLFVQDKEVATLIENLYSKLKLMLLKKDDVSINSLLSHLRKINLHLEKKNTRFLTGDTMCCFDCELMPRLQHIRVAGKYFMDFQMPTDLRYLWRYMLHMYQLDAFTQSCPADQDIVNHYKQQQNVKMKKHEELETPTFTTSIPVELDLQQDE

>*Btab*CLIC

MSEEINENGTGNGQDVPEIELIIKASTIDGRRKGACLFCQEYFMDLFLLAELKTISLKVTTVDMQKPPPDFRTNFEATHPPILIDNGLAVLENEKIERHIMKNVPGGHNLFVQDKQVATLIENLYSRLKLMLLKKDDSSTNALLSHLRKIDEHLGKTGTRFLTGDTMCCFDCELMPRLQHIRVAGKYFMEFEIPTSLTYLWRYMLHMYQLDAFTQSCPADQDIINHYKLQQGMKMKKHEELETPTFTTSIPIDISASD

>*Mp*CLIC

MADEDLPHANGNGLEENGHVPEIELIIKASTIDGRRKGACLFCQEYFMDLYLLAELKTISLKVTTVDMQKPPPDFRSNFDANPPPILIDNGMAVLENEKIERHIMKNVPGGYNLFVQDKEVATLIENLYSKLKLMLLKKDDVSINSLLSHLRKINLHLEKKNTRFLTGDTMCCFDCELMPRLQHIRVAGKYFMDFQMPTDLRYLWRYMLHMYQLDAFTQSCPADQDIVNHYKQQQNVKMKKHEELETPTFTTSIPVELDLQQDE

>*Sfla*CLIC

MADDDTAHTNGNGHEENGLVPEIELIIKASTIDGRRKGACLFCQEYFMDLYLLAELKTISLKVTTVDMQKPPPDFRSNFDANPPPILIDNGMAVLENEKIERHIMKNIPGGYNLFVQDKEVATLIENLYSKLKLMLLKKDDVSINSLLSHLRKINSHLEKKNTRFLTGDTMCCFDCELMPRLQHIRVAGKYFMDFQMPTDLRYLWRYMLHMYQLDAFTQSCPADQDIVNHYKQQQNVKMKKHEELETPTFTTSIPVELDLQDE

>*Bt*CLIC6-like

MSPCAKLCVLALLACAVNSAPVPEEESNTQLPESGPEGAVLSSDGFEGALVPSDAQVTKRQAEEAAVDDAQTPEGQGLEAGQNSGAFFAPEQGAQDPATLTQESSQFNAEAAIPQGQEASVTKRQAEEAAVDDAQTPEGQGLEAGQNSGAFFAPEQGAQDPATLTQESSQFNAEAAIPQGQEASVSKRQADTAQVEGQETSQQDAQFVQPEGQVGSANDESQVAESSLPQQEVTSVSKRQVEQPAEVPQADGQPAEVPPADEQPAEVPQADGQPAEVPQADEQPAEVPQADGQSISAPEGQFFAPLNQEAQSPAEPTGLTQDDSQVAAEGSIPQEGQTVAKRQVQEAQSADAGQAPIVPDLESQSFEGAAVEPQADAPAEIPTQDEVSQGAEDAQSKLTKRQISPDPYNVEVLANKDRKEPCVVGGDVENCDGFQAGLRQAKSASTTTDTE

>*Bk*CLIC6-like

MYSRFLSHFPEQHDFLDWHFSPFGRLGPSVWTHDSGGGKFVDPVVRFDTGDPDVTSDPVAAGGTGDAVDTGGSVDPVSTVDDGDSVETIGFVDAGDTVDPVDAGDTGDPVDTGGTVAPVSTVDAGDSVETSGFVDTGDIVDPVDAGDTGDPVDTGGTVDPVSTVDAGDSFETSGFVDTGDIVDAGDTGDPVDTGGTVGPVSTVDPVASVDPFDTGGNVDSVSTADPVETRGTVDPEATGDPVDTGRTVDPVVTVDPIDTGGTVDSVATGDPADSGCTVDPVSTGDTVDPVSTGNTGDSAGNGDAPAARRR

>*Bk*CLIC

MADDGHENGTTNGDVPEIELIIKASTIDGRRKGACLFCQEYFMDLYLLAELKTISLKVTTVDMQKPPPDFRTNFQATPPPILIDNGDAILENEKIERHIMKNIPGGHNLFVQDKEVATLVENIFSKLKLLLINSKDKEMDPKSSSLMTHLRKIDEHLGRKGTRFLTGDTMCCFDCELMPRLQHIRVAGKYFVDFEIPETLVNLWRYMHHMYRLDAFLQSCPADQDIINHYKLQQSMKLKKHEELETPTFTTSIPIEVSDD

>*Ds*CLIC6-like

MTSRKTISLGILLLLLVNLAAIEAQEGEETTAGEAAATEAPEGDAAKTEATAGEGAPGETTAGEGAPGETPAGEGAPGETTAADGAPGETTTGAASETAVGDGAPGYRRGRHSRCRWSDDREACSWWRCGRVDCSQWRTSKEKPAEEGSPTEKPAEGAKPTGDSGEVENVPALGAEQGDAGNQTTTDGSKAAPDSSKGAAEDEKDSKEEKAEKGEEDTTAKDPFYYGNGSPTTLDVAQTPGTNPTLPPETTTTTGIMDLMTYMPEDNAGDPFVSPGAAHRPGSRHVKAHDGFHSLTTEQYWAHWNDRFTTKKSRNGHHWRDLMQ

>*Ds*CLIC

MSSADVETQPQQEATNGSSKFDVPEIELIIKASTIDGRRKGACLFCQEYFMDLYLLAELKTISLKVTTVDMQKPPPDFRTNFEATHPPILIDNGLAILENEKIERHIMKNIPGGYNLFVQDKEVATLIENLYVKLKLMLVKKDDAKNNALLSHLKKINDHLANRNTRFLTGDTMCCFDCELMPRLQHIRVAGKYFVDFEIPTHLTALWRYMYHMYQLDAFTQSCPADQDIINHYKLQQSLKMKKHEELETPTFTTSIPIDISE

>*Sg*CLIC5-like

MASHNSSDNQSTPFMTLYVRAGRDGRSLGACPDSQRSIFLAELKSLSGDTEPPLKYRIIPVCPSRPPPEFTCLGEGGARLRLPAAKLDAGPWGPESVVDSADDITALLEKYFPGGVAKAATPELEAAAELATRDMFSRFCYLVRGVVRDTSQLEAELRKADAHLLGLRGPFLTGAAPTLLDVEVLPKLHQARVAAHTIRGYEIPAELTGLWRWLHAAYSLPAFVASCPCDAEITLHWLDALGESTASPERRRQMRALYEGGPRFSLTVPVRATPVVIE

>*Sg*CLIC

MADEYTNGAPENGNVPEIELIIKASTIDGRRKGACLFCQEYFMDLYLLAELKTISLKVTTVDMQKPPPDFRTNFEATPPPILIDNGLAVLENEKIERHIMKNVPGGHNLFVQDKEVATLIENLYSKLKLMLIKKDSTSTHSLLSHLRKINDHLARKGSRFLTGDTMCCFDCELMPRLQHIRVAGKYFTDFEIPMELTALWRYMYHMYQLDAFTQSCPADQDIINYYKQHKVETNKPGAMKMKKHEELETPTFTTSIPAEISLED

>*Zn*CLIC2-like

MSQSPTNISIFVKAGKDGKSLGACPQSQRVLMLAELKFAAKVLPQFKTIPVNVSRSPEAFRRLGLRLRVPALYLGLGEDPVDVADDIVSLLESRFPGGILQQNEEDPEAELVTRDFFSRFCFYVRDVSKDASHLESELRRIDCYLKNTSNNVFLCGPAPSFLDCEVLPKLHQVRVAAAGIKGYEIPANLTGLWRYLHAAYSEPAFVHTCPSDAEILLHWFDHIGESSANIRKQQHQVLNGKMPHYSFSVPVFAKPVILE

>*Zn*CLIC

MADESIQNGTNGGDVPEIELIIKASTIDGRRKGACLFCQEYFMDLYLLAELKTISLKVTTVDMQKPPPDFRTNFEATPPPILIDNGLAVLENEKIERHIMKNVPGGHNLFVQDKEVATLVENLYGKLKLMLLKKDDTKTNSLMSHLRKINEHLGRKGTRFLTGDTMCCFDCELMPRLQHIRVAGKYFLDFEIPVELEHLWHYMYHMYQLDAFTQSCPADQDIINHYKLQQGMKMKKHEELETPTFTTSIPVSVATED

>*Nl*CLIC2-like

MSTKDKVTLFYKAGYDDKCFGACYQSQRVLILSELKFSKHLMPEFTCIPVNISRPPDSFQRLGLRLRVPALHIQNELDDKIEPIDVADDIVSNLQSRFPGGVISNHWEAQAENVTSNFFSKFCYLIRGIAKDSQHLEQELSLIDEHLSNVASESDDDTGLLFLCGNQMSLIDCEVLPKLHQVRVAAASLKGKLFTFSKLL

>*Fo*CLIC-exl-1

MMNVESEDEIVLFVKLGYDGHSLGACHQSQRVNMLSRLKATAQVMPSPRVVPVNTAKPPKEFSDLDLRLRLPVLHIILATETDGLEPLDSSDDIVVALESKYPGGLCACSSQCEAEADQASRDFFSKFCFLVRGVAKESSHLESELQTLDKFLQDSSGERGEGGFLCGSSPSLLDCEILPKLHQVRVAAQALKGYSIPAHFSALWRYLYAAYSCPAFTESCPPDHEIILHWLEHCGTPEAMKQSRKLLSQIGSERHQPSYSFSIPAKATPVVIDD

>*Fo*CLIC

MAEEAVQNGNNHTDIPEIELIIKASTIDGRRKGACLFCQEYFMDLYLLAELKTVCLKVTTVDMQKPPPDFRTNFEATPPPILIDNGLAVLENEKIERHIMKNVPGGHNLFVQDKEVATLIENLYSKLKLMLLKKDEVSTNNLMSHLRKINAHLQRNNQRFLTGDTMCCFDCELMPRLQHIRVAGKYFLDFDIPVELAALWRYMYHMYQLDAFTQSCPADQDIINHYKLQQGMKMKKHEELETPTFTTSIPVGVSIDDTVN

>*Hv*CLIC-exl-1

MIAEIKFNAGVLPKYEVIPVNVSKPPKEFKELGLHLRVPALFLSLGKKEEFEPIDIADDIVLELESRYPGGLLKNELEAEAESATRNLFSKFCHWMRGVAKDSGHLETELAHLDSHLSAVSVNYPQADKLYLCGDRLALVDCEVLPKLHQVRVAAQEIKGYELPRNLTHVWRYLHSAYSDPAFVKCCPSDTEILLHWLDTGSVKLSARQQQLLAASSGKPPTFSFTVPATATRVELE

>*Cs*CLIC-exl-1

MNQSSNLTLFVKAGKDEKCLGACPQSHRVLMLAELKVAAKILPQFKTIPVNVSRPPEVFSRLGLRLRVPALYLGLDEDPIDVADDIVSLLESRFPGGILQQNQEDPEAELVTRDFFSRFCFYVRGVAKDGSQLESELRRIDFYLQNASSHGSGNSLFLCGPRPSLLDCEVLPKLHQVRVAAAGIKGYEIPSNLTGLWRYLHSAYAEPAFVRTCPSDAEILLHWLEHIGESPANIRKQQHLVLNEKIPQYSFSVPVFAKPVVLE

>*Csec*CLIC

MADESIQNGTNGGDVPEIELIIKASTIDGRRKGACLFCQEYFMDLYLLAELKTISLKVTTVDMQKPPPDFRTNFEATPPPILIDNGLAVLENEKIERHIMKNVPGGHNLFVQDKEVATLVENLYSKLKLMLLKKDDTKTNSLMSHLRKINEHLGRKGTRFLTGDTMCCFDCELMPRLQHIRVAGKYFLDFDIPVELAHLWRYMYHMYQLDAFTQSCPADQDIINHYKLQQGMKMKKHEELETPTFTTSIPVSIATED

>*Ph*CLIC-exl-1

MCFFKVASTIDGRRKGACLFCQEYFMDLYLLAELKTISLKVTTVDMQKPPPDFRTNFEATPPPILIDGGLAVLENDKIERHIMKNIPGGHNLFVQDKEVATLIENLYSKLKLALIKKEEGKPTALLTHLRKINEHLAKKDTRFLTGDTMCCFDCELMPRLQHIRVAGKYFVDFEIPGELTALWRYMYHMYQLDAFTQSCPADQDIINHYKLQIGMKMKKHEELETPTFTTSIPVEISLED

>*Lg*CLIC

MSDEIAENGTANGDVPEIELIIKASTIDGRRKGACLFCQEYFMDLYLLAELKTISLKVTTVDMQKPPPDFRTNFEATHPPILIDNGLAILENEKIERHIMKSVPGGHNLFVQDKEVASLIENLYSKLKLVLVRKDEQKSAALRAHLARIDAQLERRGTRFLTGDTMCCFDCELMPRLQHIRVAGKYFVDFEIPTSLVSLWRYMYHMYQLDAFTQSCPADQDIINHYKLQQALKMKKHEELETPTFTTSIPVDVNSHHEQ

>*Ls*CLIC

MSDEIAENGTANGDVPEIELIIKASTIDGRRKGACLFCQEYFMDLYLLAELKTISLKVTTVDMQKPPPDFRTNFEATHPPILIDNGLAILENEKIERHIMKSVPGGHNLFVQDKEVASLIENLYSKLKLVLVRKDEQKSAALRAHLGRIDALLERRGTRFLTGDTMCCFDCELMPRLQHIRVAGKYFVDFEIPADFRALWRYMFHMYQLDAFTQSCPADQDIINHYKLQQALKMKKHEELETPTFTTSIPIDINETNNGDE

>*Mj*CLIC

MSDEIAENGTANGDVPEIELIIKASTIDGRRKGACLFCQEYFMDLYLLAELKTISLKVTTVDMQKPPPDFRTNFEATHPPILIDNGLAILENEKIERHIMKSVPGGHNLFVQDKEVASLIENLYSKLKLVLVRKDEQKSAALRAHLGRIDALLERRGTRFLTGDTMCCFDCELMPRLQHIRVAGKYFVDFEIPTSFRALWRYMYHMYQLDAFTQSCPADQDIINHYKLQQALKMKKHEELETPTFTTSIPIDVNEHNHSEQ

>*Ccro*CLIC

MSDEIAENGTANGDVPEIELIIKASTIDGRRKGACLFCQEYFMDLYLLAELKTISLKVTTVDMQKPPPDFRTNFEATHPPILIDNGLAILENEKIERHIMKSVPGGHNLFVQDKEVASLIENLYSKLKLVLVRKDEQKSASLRAHLGRIDGLLERRGTRFLTGDTMCCFDCELMPRLQHIRVAGKYFVDFEIPTSFRALWRYMYHMYQLDAFTQSCPADQDIINHYKLQQALKMKKHEELETPTFTTSIPIDINDLNNAEE

>*Aage*CLIC

MSDEIAENGTANGDVPEIELIIKASTIDGRRKGACLFCQEYFMDLYLLAELKTISLKVTTVDMQKPPPDFRTNFEATHPPILIDNGLAILENEKIERHIMKSVPGGHNLFVQDKEVASLIENLYSKLKLVLVRKDEQKSASLRAHLSRIDGLLERRGTRFLTGDTMCCFDCELMPRLQHIRVAGKYFVDFEIPTSFRALWRYMYHMYQLDAFTQSCPADQDIINHYKLQQALKMKKHEELETPTFTTSIPIDVNDLNNSEQ

>*Vcar*CLIC

MMSDEIAENGTANGDVPEIELIIKASTIDGRRKGACLFCQEYFMDLYLLAELKTISLKVTTVDMQKPPPDFRTNFEATHPPILIDNGLAILENEKIERHIMKSVPGGHNLFVQDKEVASLIENLYSKLKLVLVRKDEQKSASLRAHLQRIDALLERRGTRFLTGDTMCCFDCELMPRLQHIRVAGKYFVDFEIPTSFRALWRYMYHMYQLDAFTQSCPADQDIINHYKLQQALKMKKHEELETPTFTTSIPIDVNDSNNSEQ

>*Pg*CLIC

MSDEIAENGTTNGDVPEIELIIKASTIDGRRKGACLFCQEYFMDLYLLAELKTISLKVTTVDMQKPPPDFRTNFEATHPPILIDNGLAILENEKIERHIMKSVPGGHNLFVQDKEVASLIENLYSKLKLVLVRKDEQKSSALRAHLGRIDALLERRATRFLTGDTMCCFDCELMPRLQHIRVAGKYFVDFEIPTSFRALWRYMYHMYQLDAFTQSCPADQDIINHYKLQQALKMKKHEELETPTFTTSIPVDVGDLNHAE

# Supplementary Figures and Tables

## Supplementary Figures


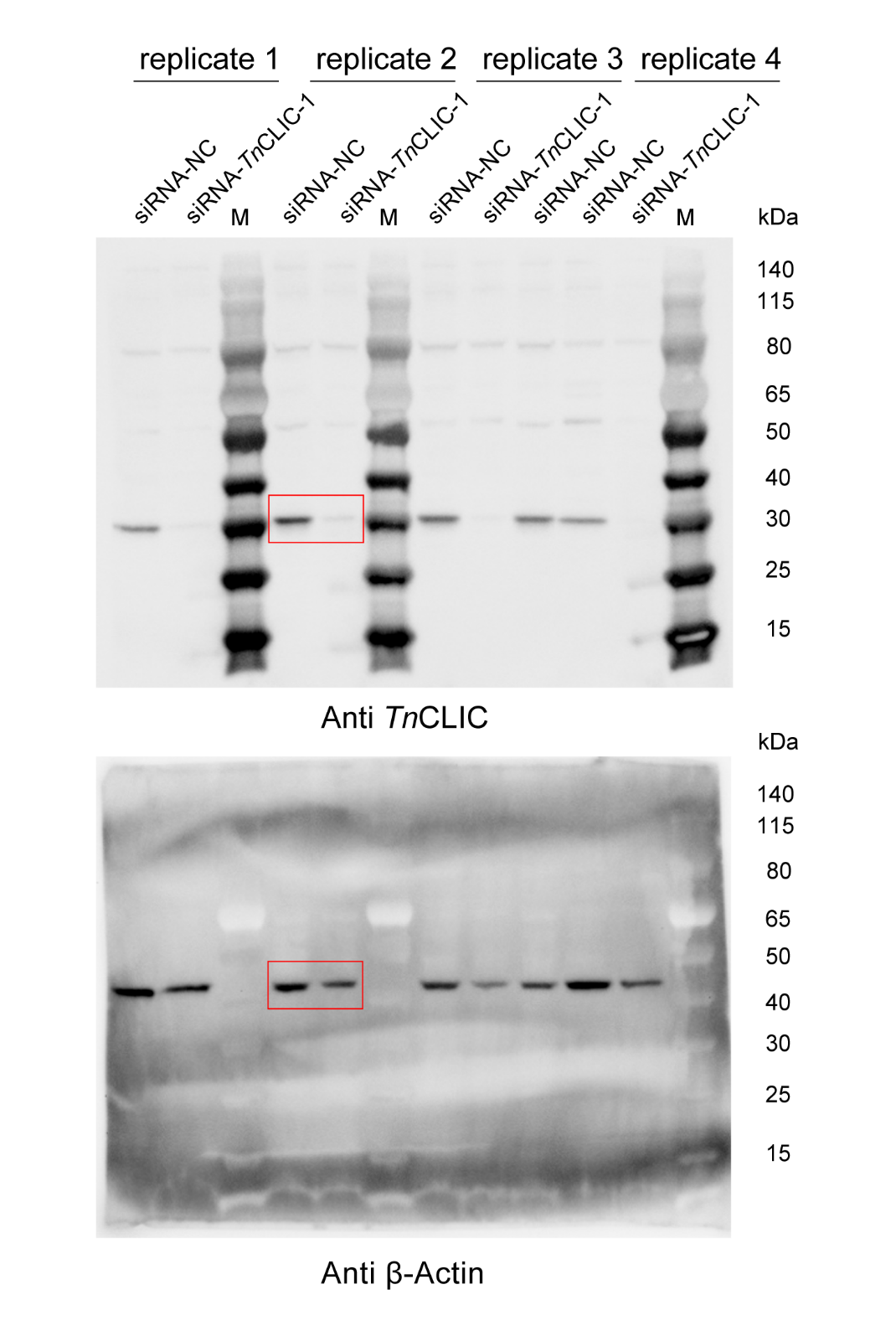


**Supplementary Figure 1.** Western blot that shows all bands with all molecular weight markers of *Tn*CLIC protein levels at 48 h after treatment, β-actin was used as a loading control. The bounding box of the example in Figure 2B is shown in Figure S1 marked with a red box. Original pictures were uploaded to the Supplementary Materials, named Figure S1_1 and Figure S1_2.


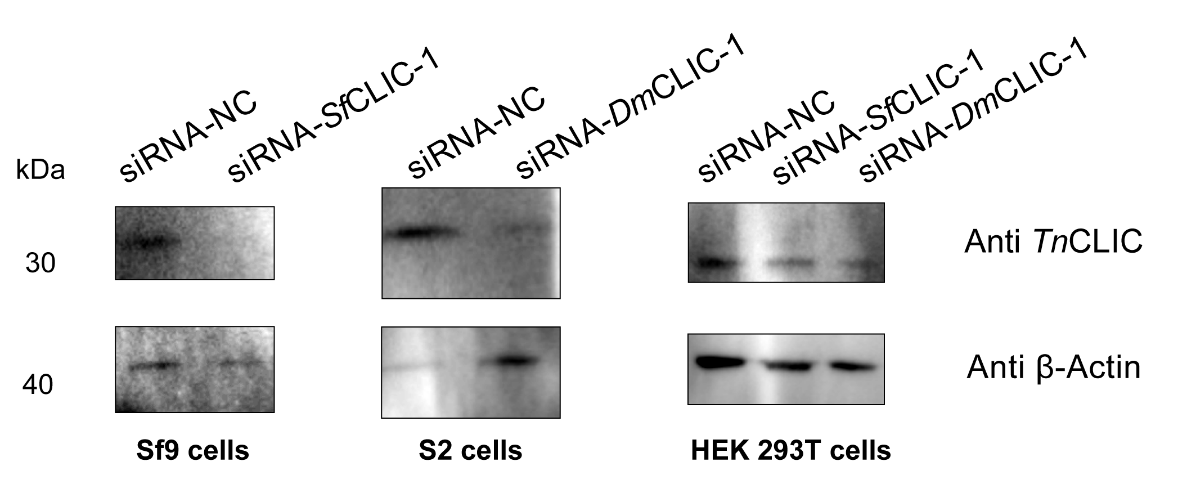


**Supplementary Figure 2.** Western blot of *Sf*CLIC, *Dm*CLIC and human CLIC4 protein levels at 48 h after treatment, β-actin was used as a loading control.


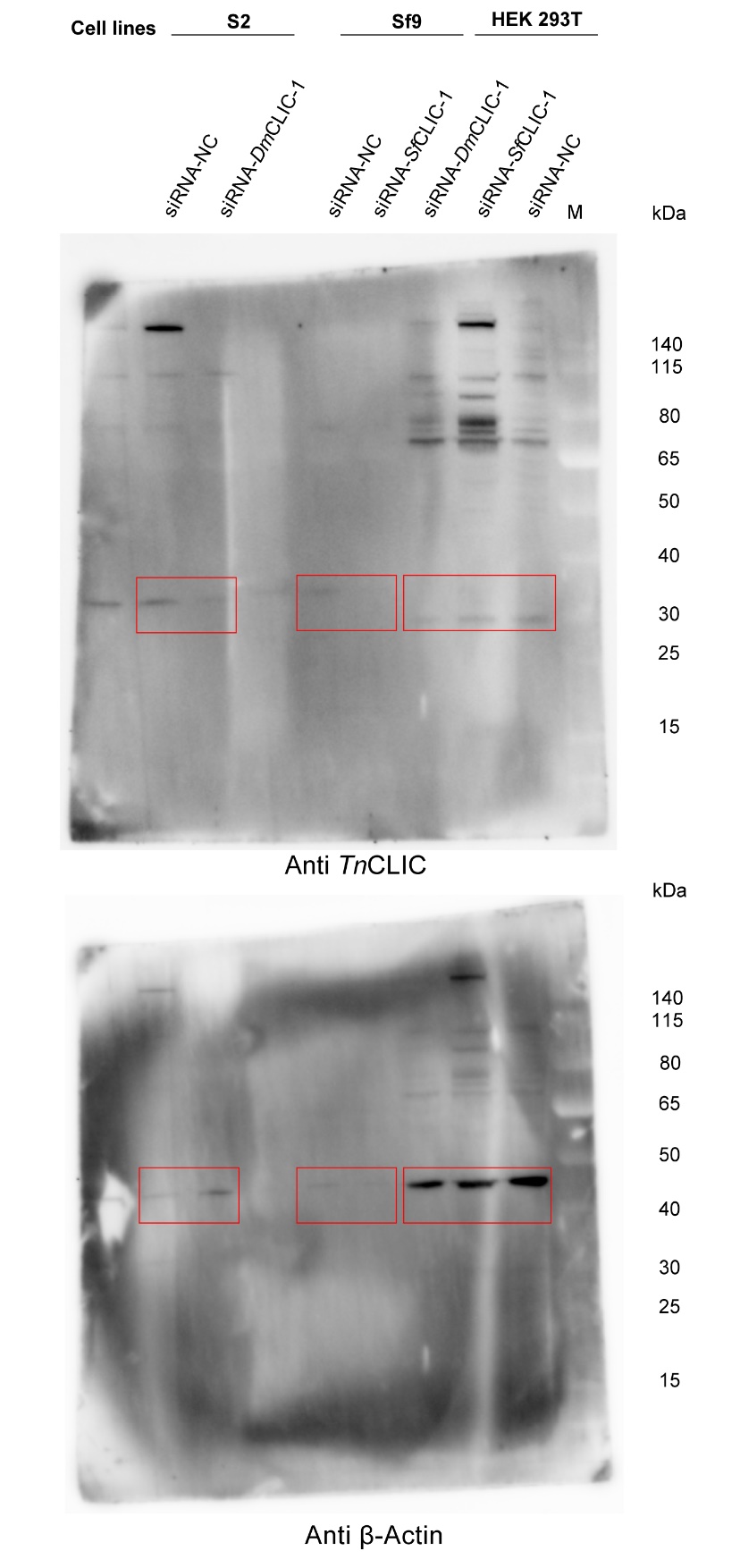


**Supplementary Figure 3.** Western blot that shows all bands with all molecular weight markers of *Sf*CLIC, *Dm*CLIC and human CLIC4 protein levels at 48 h after treatment, β-actin was used as a loading control. The bounding box of the example in Figure S2 is shown in Figure S3 marked with a red box. Original pictures were uploaded to the Supplementary Materials, named Figure S3_1 and Figure S3_2.


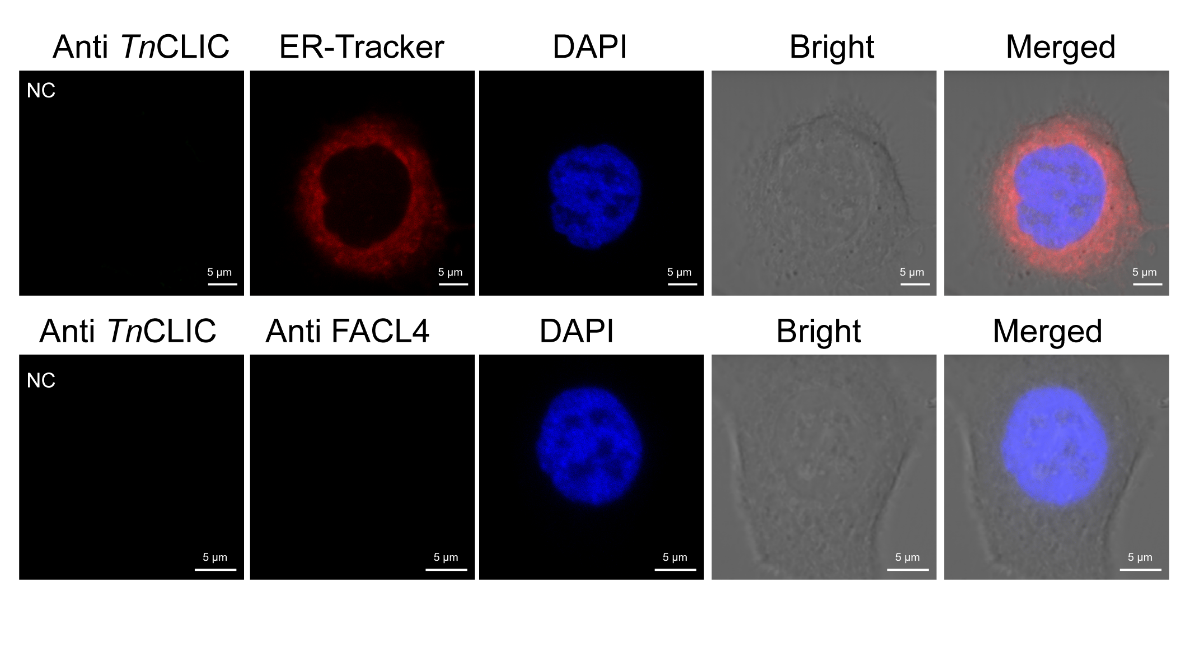


**Supplementary Figure 4.** The negative control group about *Tn*CLIC sublocalization in the endoplasmic reticulum and colocalization with fatty acid CoA ligase 4 (FACL4). NC, negative control, means Hi-5 cells without the primary antibody. Bar = 5 µm.

## Supplementary Tables

**Table S1. Primers and siRNAs used in this study**

| **Usage** | **Name** | **Sequence (5'- 3')** |
| --- | --- | --- |
| Cloning and expression | pCold-I-*Tn*CLIC-F | aggcatatggagctcggtaccATGTCGGATGAAATTGCGGA |
|  | pCold-I-*Tn*CLIC-R | agcagagattacctatctagaTTAATGATGATGATGATGATGCTCGGCATTACTGTTC |
|  | pCold-I-GFP-F | aggcatatggagctcggtaccATGGTGAGCAAGGGCGAGG |
|  | pCold-I-GFP-R | agcagagattacctatctagaTTAATGATGATGATGATGATGCTTGTACAGCTCGTCCATGCC |
| Transfection | siRNA-*Sf*/*Tn*CLIC-1-F | GCAAGUACUUCGUCGACUUTT |
|  | siRNA-*Sf*/*Tn*CLIC-1-R | AAGUCGACGAAGUACUUGCTT |
|  | siRNA-*Tn*CLIC-2-F | GCCUCGCAAUACUGGAGAATT |
|  | siRNA-*Tn*CLIC-2-R | UUCUCCAGUAUUGCGAGGCTT |
|  | siRNA-*Sf*CLIC-2-F | GCAGAGGGACUAGAUUCUUTT |
|  | siRNA-*Sf*CLIC-2-R | AAGAAUCUAGUCCCUCUGCTT |
|  | siRNA-*Dm*CLIC-1-F | GCAAGUACUUUGUCGACUUTT |
|  | siRNA-*Dm*CLIC-1-R | AAGUCGACAAAGUACUUGCTT |
|  | siRNA-*Dm*CLIC-2-F | CCACGUUUACCACAUACAUTT |
|  | siRNA-*Dm*CLIC-2-R | AUGUAUGUGGUAAACGUGGTT |
|  | siRNA-NC-F | UUCUCCGAACGUGUCACGUTT |
|  | siRNA-NC-R | ACGUGACACGUUCGGAGAATT |
| qPCR | qPCR-*Tn*CLIC-F | CTCTGGCGCTACATGTACCA |
|  | qPCR-*Tn*CLIC-R | CGGCATTACTGTTCTCGCTC |
|  | qPCR-*Tn*-RPS5-F | CGACAGCATGCCTTTACCGC |
|  | qPCR-*Tn*-RPS5-R | CATCCACAGCCTGACGACGC |
|  | qPCR-*Sf*CLIC-F | TTTCCGCACCAACTTCGAAG |
|  | qPCR-*Sf*CLIC-R | CCGACTTCTGCTCGTCTTTG |
|  | qPCR-*Sf*-actin-F | GATCTGGCACCACACCTTCT |
|  | qPCR-*Sf*-actin-R | GGCGTGTTGAAGGTCTCGAA |
|  | qPCR-*Dm*CLIC-F | GACGAACGGCAGCAGTAAAT |
|  | qPCR-*Dm*CLIC-R | GGGTGTGTGGCCTCAAAATT |
|  | qPCR-*Dm*-RPL32-F | CCCAAGATCGTGAAGAAGCG |
|  | qPCR-*Dm*-RPL32-R | GCTTGTTCGATCCGTAACCG |
|  | qPCR-*Hs*CLIC4-F | AAGGAGGAGGACAAAGAGCC |
|  | qPCR-*Hs*CLIC4-R | GGTCTGCTGGCTTCCTTTTC |
|  | qPCR-*Hs*-GAPDH-F | GACAAGCTTCCCGTTCTCAG |
|  | qPCR-*Hs*-GAPDH-R | GAGTCAACGGATTTGGTGGT |
|  | LOC113491855-F | ACCCAACACTTTGCAAGGAC |
|  | LOC113491855-R | GCTCCTGGTTCCAATGTTGT |
|  | LOC113503987-F | CCTCGCGCACTACTACATCA |

**Continued Table S1**

| **Usage** | **Name** | **Sequence (5'- 3')** |
| --- | --- | --- |
| qPCR | LOC113503987-R | CGCGTAGATCACGTCTTTGA |
|  | LOC113492230-F | GATGCTGAGGCTATGCAACA |
|  | LOC113492230-R | GGAGCCGTCAGTGTCAATTT |
|  | LOC113498006-F | TTGTCCGATGTGGAGATGAA |
|  | LOC113498006-R | GCATAAGGGATGAAGCCAAA |
|  | LOC113493155-F | ACAGGGCTGTGCTTGAGAAT |
|  | LOC113493155-R | AGCTGTTGGAAGTGGAAGGA |
|  | LOC113492377-F | AGTGGCAGACTCAGTTTCGA |
|  | LOC113492377-R | TTCATCAGAAAGGCGCCAAC |
|  | LOC113494039-F | CAACGACGGGTACATCACAC |
|  | LOC113494039-R | GTGAGAGAGCCTGCACAATG |
|  | LOC113494243-F | TTGACAACCCTCTCGAGTCC |
|  | LOC113494243-R | CGGCCATCTTTGTTCGAGTC |
|  | LOC113502764-F | CTCCAGGATGGAATGAGGAA |
|  | LOC113502764-R | GGATATCATGAGGGCGAAGA |
|  | LOC113502137-F | AAGAAGAAAGGGCACAAGCA |
|  | LOC113502137-R | CCACCTCCTTCACCATGTCT |
|  | LOC113500453-F | GGCTTTGCCAGAGATGTAGC |
|  | LOC113500453-R | AGGGTGACAATCTCCCACAG |

**Supplementary Table 2. Insect CLIC for phylogenetic analysis**

| **Order** | **Species** | **Abbreviation** | **CLIC (number)** |
| --- | --- | --- | --- |
| Hemiptera | *Acyrthosiphon pisum* | *Apis* | CLIC (1) |
| Hemiptera | *Aphis gossypii* | *Ag* | CLIC (1) |
| Hemiptera | *Bemisia tabaci* | *Btab* | CLIC (1)  CLIC6-like (1) |
| Hemiptera | *Homalodisca vitripennis* | *Hv* | CLIC-exl-1 (1) |
| Hemiptera | *Myzus persicae* | *Mp* | CLIC (1) |
| Hemiptera | *Nilaparvata lugens* | *Nlug* | CLIC2-like (1)  CLIC (1) |
| Hemiptera | *Sipha flava* | *Sfla* | CLIC (1) |
| Coleoptera | *Aethina tumida* | *Atum* | CLIC (1) |
| Coleoptera | *Agrilus planipennis* | *Apla* | CLIC (1) |
| Coleoptera | *Dendroctonus ponderosae* | *Dpon* | CLIC (1) |
| Coleoptera | *Diabrotica virgifera* | *Dv* | CLIC (1) |
| Coleoptera | *Harmonia axyridis* | *Haxy* | CLIC (1) |
| Coleoptera | *Leptinotarsa decemlineata* | *Ld* | CLIC (1) |
| Coleoptera | *Nicrophorus vespilloides* | *Nves* | CLIC (1) |
| Coleoptera | *Onthophagus taurus* | *Ot* | CLIC (1) |
| Coleoptera | *Sitophilus oryzae* | *So* | CLIC (1) |
| Coleoptera | *Tribolium castaneum* | *Tc* | CLIC (1) |
| Hymenoptera | *Acromyrmex echinatior* | *Ae* | CLIC (1) |
| Hymenoptera | *Anastatus fulloi* | *Af* | CLIC (1) |
| Hymenoptera | *Anastatus japonicus* | *Aj* | CLIC (1) |
| Hymenoptera | *Anisopteromalus calandrae* | *Acal* | CLIC (1) |
| Hymenoptera | *Apis cerana* | *Acer* | CLIC (1) |
| Hymenoptera | *Athalia rosae* | *Ar* | CLIC (1) |
| Hymenoptera | *Atta cephalotes* | *Acep* | CLIC (1) |

**Continued Supplementary Table 2**

| **Order** | **Species** | **Abbreviation** | **CLIC (number)** |
| --- | --- | --- | --- |
| Hymenoptera | *Belonocnema kinseyi* | *Bk* | CLIC (1)  CLIC6-like (1) |
| Hymenoptera | *Belonocnema treatae* | *Btre* | CLIC (1) |
| Hymenoptera | *Bombus impatiens* | *Bi* | CLIC (1) |
| Hymenoptera | *Bombus terrestris* | *Bter* | CLIC (1) |
| Hymenoptera | *Cephus cinctus* | *Ccin* | CLIC (1) |
| Hymenoptera | *Ceratosolen solmsi marchali* | *Csol* | CLIC (1) |
| Hymenoptera | *Chelonus insularis* | *Ci* | CLIC (1) |
| Hymenoptera | *Copidosoma floridanum* | *Cf* | CLIC (1) |
| Hymenoptera | *Cotesia chilonis* | *Cchi* | CLIC (1) |
| Hymenoptera | *Cotesia vestalis* | *Cv* | CLIC (1) |
| Hymenoptera | *Diachasma alloeum* | *Da* | CLIC (1) |
| Hymenoptera | *Dufourea novaeangliae* | *Dn* | CLIC (1) |
| Hymenoptera | *Eufriesea mexicana* | *Em* | CLIC (1) |
| Hymenoptera | *Fopius arisanus* | *Fa* | CLIC (1) |
| Hymenoptera | *Formica exsecta* | *Fe* | CLIC (1) |
| Hymenoptera | *Gonatopus flavifemur* | *Gfla* | CLIC (1) |
| Hymenoptera | *Habrobracon hebetor* | *Hh* | CLIC (1) |
| Hymenoptera | *Habropoda laboriosa* | *Hl* | CLIC (1) |
| Hymenoptera | *Lysiphlebus fabarum* | *Lf* | CLIC (1) |
| Hymenoptera | *Macrocentrus cingulum* | *Macin* | CLIC (1) |
| Hymenoptera | *Megachile rotundata* | *Mr* | CLIC (1) |
| Hymenoptera | *Microplitis demolitor* | *Mdem* | CLIC (1) |
| Hymenoptera | *Nasonia vitripennis* | *Nvit* | CLIC (1) |
| Hymenoptera | *Neodiprion lecontei* | *Nlec* | CLIC (1) |
| Hymenoptera | *Ooceraea biroi* | *Ob* | CLIC (1) |
| Hymenoptera | *Orussus abietinus* | *Oa* | CLIC (1) |

**Continued Supplementary Table 2**

| **Order** | **Species** | **Abbreviation** | **CLIC (number)** |
| --- | --- | --- | --- |
| Hymenoptera | *Pachycrepoideus vindemmiae* | *Pvin* | CLIC (1) |
| Hymenoptera | *Pogonomyrmex barbatus* | *Pb* | CLIC (1) |
| Hymenoptera | *Polistes canadensis* | *Pc* | CLIC (1) |
| Hymenoptera | *Polistes dominula* | *Pd* | CLIC (1) |
| Hymenoptera | *Pteromalus puparum* | *Ppup* | CLIC (1) |
| Hymenoptera | *Pteromalus qinghaiensis* | *Pq* | CLIC (1) |
| Hymenoptera | *Pteromalus venustus* | *Pven* | CLIC (1) |
| Hymenoptera | *Solenopsis invicta* | *Si* | CLIC (1) |
| Hymenoptera | *Telenomus remus* | *Tr* | CLIC (1) |
| Hymenoptera | *Theocolax elegans* | *Te* | CLIC (1) |
| Hymenoptera | *Trichogramma pretiosum* | *Tp* | CLIC (1) |
| Hymenoptera | *Venturia canescens* | *Vcan* | CLIC (1) |
| Hymenoptera | *Vespa mandarinia* | *Vm* | CLIC (1) |
| Lepidoptera | *Aricia agestis* | *Aage* | CLIC (1) |
| Lepidoptera | *Amyelois transitella* | *Atra* | CLIC (1) |
| Lepidoptera | *Bicyclus anynana* | *Ba* | CLIC (1) |
| Lepidoptera | *Bombyx mandarina* | *Bman* | CLIC (1) |
| Lepidoptera | *Bombyx mori* | *Bmor* | CLIC (1) |
| Lepidoptera | *Colias croceus* | *Ccro* | CLIC (1) |
| Lepidoptera | *Danaus plexippus* | *Dple* | CLIC (1) |
| Lepidoptera | *Galleria mellonella* | *Gm* | CLIC (1) |
| Lepidoptera | *Heliconius melpomene* | *Hm* | CLIC (1) |
| Lepidoptera | *Helicoverpa armigera* | *Harm* | CLIC (1) |
| Lepidoptera | *Helicoverpa zea* | *Hz* | CLIC (1) |
| Lepidoptera | *Hyposmocoma kahamanoa* | *Hk* | CLIC (1) |
| Lepidoptera | *Leguminivora glycinivorella* | *Lg* | CLIC (1) |
| Lepidoptera | *Leptidea sinapis* | *Ls* | CLIC (1) |

**Continued Supplementary Table 2**

| **Order** | **Species** | **Abbreviation** | **CLIC (number)** |
| --- | --- | --- | --- |
| Lepidoptera | *Maniola jurtina* | *Mj* | CLIC (1) |
| Lepidoptera | *Manduca sexta* | *Ms* | CLIC (1) |
| Lepidoptera | *Melitaea cinxia* | *Mecin* | CLIC (1) |
| Lepidoptera | *Papilio machaon* | *Pm* | CLIC (1) |
| Lepidoptera | *Papilio polytes* | *Ppol* | CLIC (1) |
| Lepidoptera | *Papilio xuthus* | *Pxut* | CLIC (1) |
| Lepidoptera | *Pararge aegeria* | *Pa* | CLIC (1) |
| Lepidoptera | *Pectinophora gossypiella* | *Pg* | CLIC (1) |
| Lepidoptera | *Pieris rapae* | *Pr* | CLIC (1) |
| Lepidoptera | *Plutella xylostella* | *Pxyl* | CLIC (1) |
| Lepidoptera | *Spodoptera frugiperda* | *Sfru* | CLIC (1) |
| Lepidoptera | *Spodoptera litura* | *Sl* | CLIC (1) |
| Lepidoptera | *Trichoplusia ni* | *Tn* | CLIC (1) |
| Lepidoptera | *Vanessa cardui* | *Vcar* | CLIC (1) |
| Diptera | *Aedes aegypti* | *Aaeg* | CLIC (1) |
| Diptera | *Bactrocera cucurbitae* | *Bc* | CLIC (1) |
| Diptera | *Bactrocera oleae* | *Bo* | CLIC (1) |
| Diptera | *Bactrocera dorsalis* | *Bd* | CLIC (1) |
| Diptera | *Ceratitis capitata* | *Ccap* | CLIC (1) |
| Diptera | *Culex pipiens* | *Cp* | CLIC (1) |
| Diptera | *Culex quinquefasciatus* | *Cq* | CLIC (1) |
| Diptera | *Drosophila melanogaster* | *Dm* | CLIC (1) |
| Diptera | *Drosophila subobscura* | *Ds* | CLIC (1)  CLIC6-like (1) |
| Diptera | *Glossina fuscipes* | *Gfus* | CLIC (1) |
| Diptera | *Hermetia illucens* | *Hi* | CLIC (1) |
| Diptera | *Musca domestica* | *Mdom* | CLIC (1) |

**Continued Supplementary Table 2**

| **Order** | **Species** | **Abbreviation** | **CLIC (number)** |
| --- | --- | --- | --- |
| Diptera | *Stomoxys calcitrans* | *Sc* | CLIC (1) |
| Diptera | *Zeugodacus cucurbitae* | *Zc* | CLIC (1) |
| Orthoptera | *Schistocerca gregaria* | *Sg* | CLIC (1)  CLIC5-like (1) |
| Isoptera | *Zootermopsis nevadensis* | *Zn* | CLIC2-like (1)  CLIC (1) |
| Isoptera | *Cryptotermes secundus* | *Csec* | CLIC-exl-1 (1)  CLIC (1) |
| Anoplura | *Pediculus humanus corporis* | *Ph* | CLIC-exl-1 (1) |
| Thysanoptera | *Frankliniella occidentalis* | *Fo* | CLIC-exl-1 (1)  CLIC (1) |
